# Multiplex base- and prime-editing with drive-and-process CRISPR arrays

**DOI:** 10.1038/s41467-022-30514-1

**Published:** 2022-05-19

**Authors:** Qichen Yuan, Xue Gao

**Affiliations:** 1 Department of Chemical and Biomolecular Engineering, Rice University, Houston, TX USA; 2 Department of Bioengineering, Rice University, Houston, TX USA; 3 Department of Chemistry, Rice University, Houston, TX USA

**Keywords:** Biotechnology, Genetics, CRISPR-Cas9 genome editing

## Abstract

Current base- and prime-editing technologies lack efficient strategies to edit multiple genomic loci simultaneously, limiting their applications in complex genomics and polygenic diseases. Here, we describe drive-and-process (DAP) CRISPR array architectures for multiplex base-editing (MBE) and multiplex prime-editing (MPE) in human cells. We leverage tRNA as the RNA polymerase III promoter to drive the expression of tandemly assembled tRNA-guide RNA (gRNA) arrays, of which the individual gRNAs are released by the cellular endogenous tRNA processing machinery. We engineer a 75-nt human cysteine tRNA (hCtRNA) for the DAP array, achieving up to 31-loci MBE and up to 3-loci MPE. By applying MBE or MPE elements for deliveries via adeno-associated virus (AAV) and lentivirus, we demonstrate simultaneous editing of multiple disease-relevant genomic loci. Our work streamlines the expression and processing of gRNAs on a single array and establishes efficient MBE and MPE strategies for biomedical research and therapeutic applications.

## Abstract (web summary)

Current base- and prime-editing technologies lack efficient strategies to edit multiple genomic loci simultaneously, limiting their applications in complex genomics and polygenic diseases. Here the authors describe drive-and-process CRISPR array architectures for multiplex base-editing and multiplex prime-editing in human cells.

## Introduction

Base editors and prime editors are high-precision genome editing tools that can be programmed to alter the desired context of the genome in living cells, without causing DNA double-strand breaks or requiring DNA donors^1–3^. DNA base editors are composed of deaminases fused to catalytically impaired nickase Cas9 (nCas9, D10A) and have enabled efficient base-pair conversions, including C•G to T•A by cytosine base editors (CBEs)^1^, A•T to G•C by adenine BEs (ABEs)^2^, C•G to G•C by CGBEs^4^, and dual-deaminase base editors^5^. Prime editors contain a nCas9 (H840A) tethered to an engineered reverse transcriptase (RT) programmed by prime editing gRNAs (pegRNAs) that encode the desired editing information for targeted insertions, deletions, or installation of all types of point mutations^6^. Recently, both base editing (BE) and prime editing (PE) have been used for research and applications in a variety of cell types and animal models^7^, e.g., using BE to correct the C•G to T•A point mutation in the Lamin A gene that causes the Hutchinson–Gilford progeria syndrome in mice^8^, applying PE in human cardiomyocytes to correct the exon 51 deletion mutation in the dystrophin gene that causes Duchenne muscular dystrophy^9^.

The ability to simultaneously edit multiple genomic loci with BEs or PEs would enable the study of complex functional genomics and the treatment of polygenic diseases. However, the multiplex editing ability of BE and PE is limited. Because the Cas9 used in those editors cannot mature its gRNAs from a single array and existing Cas9-based multiplex strategies require large expression constructs that cause delivery burdens^10,11^. Although pooling multiple gRNAs is a straightforward method^12–14^, it is not feasible when viral deliveries are needed for in vivo applications. Cas12a can catalyze the maturation of its gRNAs and has been used for multiplex gene knockouts, transcriptional regulations, and genetic perturbations^15–21^. Unlike Cas9, Cas12a lacks a nickase variant that only cuts the non-base-edited DNA strand for high BE efficiencies^22^, while Cas12a-based PEs have not yet been successfully developed. Moreover, Cas9 proteins used in BE and PE have already been widely discovered and engineered with expanded recognition of protospacer adjacent motifs (PAMs), superior editing activity, and high specificity^23–26^.

Here, we develop drive-and-process (DAP) arrays for multiplex base editing (MBE) and multiplex prime editing (MPE) in human cells. DAP architectures use tRNA itself to express tandemly assembled tRNA-gRNA array, followed by the endogenous tRNA processing machinery to release the individual gRNAs for MBE and MPE. We engineered a 75-nt human cysteine tRNA (hCtRNA) that functions efficiently in DAP arrays, achieving up to 31-loci MBE and up to 3-loci MPE with efficiencies, which are most comparable to or higher than the single-site BE and PE systems. Our MBE systems showed reduced Cas9-dependent off-target editing and do not cause higher Cas9-independent off-target editing by comparing with single-site editing. To demonstrate the therapeutic potential of our MBE and MPE systems, we designed and packaged an intein-split dual-deaminase MBE system into AAV to collectively install disease-suppressing mutations at HBG1 and HBG2 loci. We then adapted the DAP array to lentiviral delivery and packaged the MPE cassette within a single lentivirus for precise 6-bp deletion in the BCL11A gene. Together, our MBE and MPE platforms enable streamlined, scalable, and efficient multiplex precision gene editing, paving the road for the future study of polygenic diseases and complex functional genomics.

## Results

### Evaluation of dCas12a multiplex base editing strategy

**Fig. 1:**
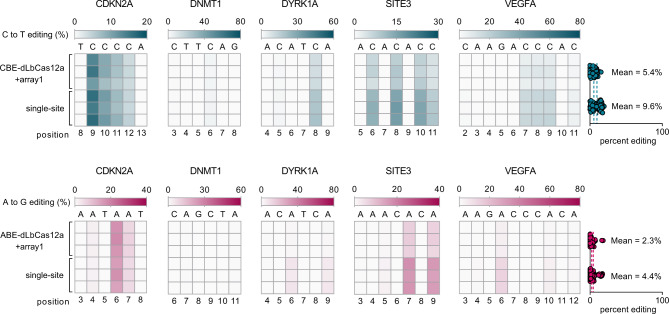
Cas12a multiplex strategy for MBE. Heat maps show 3 independent biological replicates at each condition, counting the most PAM-distal position as 1. The dashed line in scatters dot plots shows the mean value of all desired BE efficiencies within the displayed activity windows of each site. Single-site, using CBE-dCas12a or ABE-dCas12a with the relevant hU6-driven gRNA for single-site base editing. Detailed constructs of CBE-dCas12a (2xUGI-rAPOBEC1-dCas12a) and ABE-dCas12a (TadA-8e-dCas12a) are available in Supplementary Table 1. Protospacer and amplicon information can be found in Supplementary Table 5.

Although DNase dead Cas12a fused base editors (dCas12a-BE) exhibit lower editing efficiencies than nCas9-BE in a single genomic site, the multiplex gene editing potential of dCas12a-BE has yet to be evaluated. We chose dLbCas12a (LbCas12a D832A)^27^, a Cas12a variant that has been constructed as both CBE (dCpf1-BE) and ABE (LbABE8e) for single-site BEs^28–30^, and designed multiplex gRNA array for dLbCas12a-MBE under the same array architecture as for LbCas12a-nuclease based multiplex gene knockouts^29^. A human U6 (hU6) promoter was used to drive five tandemly assembled gRNAs that were validated for single-site BEs^28^. The individual gRNAs of this multiplex array can be processed and released by dLbCas12a-BE via the dedicated RNase-domain of dLbCas12a^29^. The designed 5-loci gRNA array is referred to as array1 (Supplementary Fig. 1), which was transfected with CBE-dLbCas12a or ABE-dLbCas12a (Supplementary Table 1) into HEK293T cells. We also performed single-site dCas12a-BE using CBE-dCas12a or ABE-dCas12a with hU6 promoter expressing each gRNA individually. To assess MBE activities, we chose the efficiently edited region of 6 to 10 bp for each site and calculated all desired C•G to T•A or A•T to G•C base-pair conversions. We observed generally low MBE efficiencies for both ABE-dLbCas12a (up to 18.1%, mean = 2.3%) and CBE-dLbCas12a (up to 15.4%, mean = 5.4%), slightly lower than the single-site editing efficiencies of ABE-dLbCas12a (mean = 4.4%) and CBE-dLbCas12a (mean = 9.6%) (Fig. 1), showing the necessity to develop more powerful multiplex strategies based on nCas9-BE.

### MBE using nCas9 and tRNA-gRNA multiplex arrays

To enable nCas9-based MBE, we sought to test the concept of tRNA-gRNA multiplex strategy^31^, which releases individual gRNAs from a tandemly assembled tRNA-gRNA array processed by cellular endogenous RNase P at 5′ end and RNase Z at 3′ end of the tRNA, respectively (Supplementary Fig. 2a). We reasoned that using a human origin tRNA variant with high genomic copy numbers^32^, indicating active cell usage, might enable the tRNA processing system to release gRNAs more efficiently. Using genomic tRNA database^33^, we selected eight mature tRNAs (RNase fully processed tRNA sequence)^34^, including seven from human (hCys GCA, hAla AGC, hAsn GTT, hLys CTT, hIIe AAT, hGly GCC, and hGln CTG) (13–29 copies per genome), and a previously reported tRNA from a plant source (AtGly GCC)^31^ (Supplementary Fig. 2b and Supplementary Table 2).

**Fig. 2:**
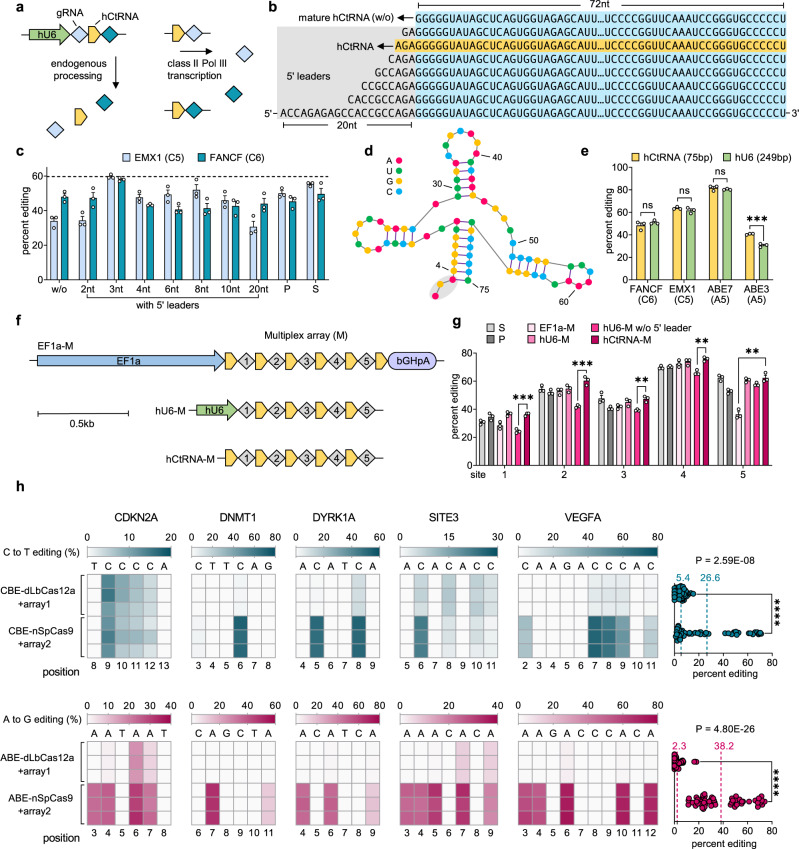
Development of DAP strategy for MBE. **a** Schematic of 2-loci hCtRNA-gRNA array driven by hU6 and using hCtRNA as Pol III promoter. hU6, human U6 promoter (RNA Pol III). **b** 5′ leader engineering of hCtRNA. **c** Adding 3 nt 5′ leader to mature hCtRNA (72 nt) showing efficient 2-loci hU6-driven MBE. P, pooled gRNAs; S, single gRNA; CBE used, NBE4max (R33A + K34A). **d**, Secondary structure of hCtRNA, gray shadow showing 3 nt 5′ leader. **e**, hCtRNA in comparison with hU6 as gRNA promoter for single-guide BE. CBE used, NBE4max (R33A + K34A); ABE used, ABE7.10 (F148A). **f**, **g** Efficient 5-loci MBE using hCtRNA-M without additional upstream RNA Pol II (EF1a) or Pol III (hU6). hU6-M w/o 5′ leader, using mature hCtRNA (72 nt) in the hU6-M array. bGHpA, bovine growth hormone poly-A termination signal. **h** Comparison of nSpCas9-MBE and dLbCas12a-MBE. Data of dLbCas12a-MBE is from Fig. 1. Heat maps show 3 independent biological replicates at each condition, counting 1 as the most PAM-distal position. Dashed line showing mean value. **c**, **e**, **g** were analyzed using Sanger sequencing, **h** was analyzed using NGS. Error bars represent mean ± s.e.m. from *n* = 3 replicates (**e**, **h** use unpaired two-tailed *t*-test; **g** uses unpaired multiple t-test; ns, not significant; *P < 0.05; **P < 0.01; ***P < 0.001; ****P < 0.0001). All tests were performed in HEK293T cells. Multiple *t*-tests information is available in Supplementary Table 4. Detailed protospacers, edited bases, and amplicons of the relevant figures are available in Supplementary Table 5.

We designed a CBE architecture by relocating the uracil glycosylase inhibitors (UGI) to the N-terminus of rAPOBEC1 cytidine deaminase, hereafter referred to as NBE4max, showing similar editing efficiencies comparing to BE4max^35^ (Supplementary Fig. 3). By replacing the BPSV40 nuclear localization signal (NLS) with a nucleoplasmin NLS, NBE4max (R33A + K34A)^36^ achieved ~1.1-fold improved BE efficiencies (Supplementary Fig. 4). We transfected HEK293T cells with NBE4max (R33A + K34A) by using the hU6 (RNA pol III promoter) to drive each selected tRNA-gRNA array of two frequently tested human genomic loci, EMX1 and FANCF sites for MBE (Fig. 2a). At the FANCF site, four tRNAs (hCys GCA, hAla AGC, hIIe AAT, and hGln CTG) enabled similar editing efficiencies (48.7 ± 3.5% (mean ± s.d.), 45.3 ± 2.1%, 48.7 ± 2.1%, and 50.3 ± 1.5%, respectively) to the pooled-gRNA delivery (P) (45.3 ± 3.8%) and single-gRNA delivery (S) (49.7 ± 5.5%). However, at the EMX1 site, all eight tRNA arrays showed lower editing efficiencies (25–35%) than those of S (55.3 ± 1.2%) or P delivery (55.3 ± 1.2%) (Supplementary Fig. 2b).

We speculated that the human endogenous RNase P failed to process the 5′ context of tRNA (3′ end of gRNA scaffold) sufficiently, which generated a gRNA with extra tRNA sequence residues at its 3′ end and adversely affected the complex formation between base editor and gRNA, thus resulting in the decreased BE efficiencies at the first EMX1 site. We hypothesized that adding the 5′ leader sequence to tRNA on the multiplex array, which is required for endogenous RNase P processing^31^, might improve the MBE efficiencies at the EMX1 locus. We further engineered three mature human tRNAs with different MBE efficiencies, including hCys GCA, hIIe AAT, and hGly GCC (Supplementary Fig. 2b). Upon adding proper lengths of 5′ leader sequences (2–20 nt), all three tRNA arrays increased the MBE efficiencies at both EMX1 and FANCF sites (Fig. 2b–d, Supplementary Fig. 2c, d). Among all tested constructs, hCys GCA with a 3 nt-5′ leader (75 bp in total length), hereafter referred to as hCtRNA, enabled the highest MBE efficiencies (averaging 59.7 ± 1.2% at EMX1; 58.0 ± 1.0% at FANCF) and multiplex knockout efficiencies (52.0 ± 3.5% at EMX1; 41.7 ± 4.0% at FANCF) (Fig. 2b–d, Supplementary Fig. 5g). Meanwhile, further truncations of the hCtRNA led to significantly decreased MBE efficiency at both the EMX1 site (averaging 37.2 ± 6.8%) and FANCF site (averaging 9.2 ± 8.4%) (Supplementary Figs. 5a–d), suggesting that the full-length hCtRNA is essential to achieve efficient multiplex editing in human cells. Next, we tested the 3- and 4-loci MBE by transfecting HEK293T cells with the designed arrays and NBE4max (R33A + K34A), observing 55.9 ± 11.7% and 53.3 ± 9.8% averaging editing efficiencies, respectively (Supplementary Figs. 5e, f). We also demonstrated that MBE using hU6-driven tRNA-gRNA architecture is sequence-specific (Supplementary Figs. 5e) and not significantly influenced by gRNA orders on the tested 3- or 4-loci array (Supplementary Figs. 5f).

### Engineer DAP arrays for MBE

We then sought to develop a compact tRNA-gRNA architecture by using the tRNA as Pol III promoter^37^ (Fig. 2a) so that no additional promoter is needed. We firstly used hCtRNA as a gRNA promoter for single-site BE in HEK293T cells and observed efficiencies similar to or higher than using hU6 promoter, with NBE4max (R33A + K34A) reaching 48.3 ± 3.8% (FANCF) and 63.7 ± 1.2% (EMX1), ABE7.10 (F148A)^38^ reaching 81.7 ± 2.3% (ABE7 site) and 40.7 ± 0.6% (ABE3 site) (Fig. 2e). We also showed that hCtRNA promoter enabled slightly higher efficiency for single-site BE than the previously reported hGln CTG tRNA promoter^39^ (Supplementary Fig. 5g).

Next, we compared three hCtRNA-gRNA constructs driven by RNA Pol II promoter (EF1a), RNA Pol III promoter (hU6), or hCtRNA itself for MBE, hereafter referred to as EF1a-M, hU6-M, and hCtRNA-M, respectively (Fig. 2f). We assembled five gRNAs into each construct and tested their efficiency using ABE7.10 (F148A) in HEK293T cells. We observed efficient MBE with hCtRNA-M (averaging 56.4 ± 15.1%) which is comparable to hU6-M (54.1 ± 14.3%) and significantly higher than EF1a-M (46.3 ± 17.0%) (Fig. 2g). Notably, deletion of the 5′ leader sequence in hU6-M led to 10-20% decreased multiplex editing efficiencies tested by both ABE7.10 (F148A) and Cas9 nuclease (Fig. 2g, Supplementary Figs. 6 and 7), demonstrating the necessity to use the 5′ leader for enhanced MBE. To unbiasedly compare the nCas9-BE using hCtRNA-M with the dCas12a-MBE strategy, we tested the MBE efficiencies of nCas9-BE variants at the same five sites (array2, Supplementary Fig. 8) as array1 (Supplementary Fig. 1). We chose the same activity window and observed up to 15-fold higher efficiencies with ABE-nSpCas9 (ABE8e, up to 74.0% with mean = 38.2) or CBE-nSpCas9 (NBE4max, up to 71.0% with mean = 26.6) than dCas12a MBE (Fig. 2h). In addition, EditR^40^ and CRISPResso2^41^ analysis of the hCtRNA-M group (Fig. 2g) were compared and no significant differences were observed (Supplementary Fig. 9). Further, we placed hCtRNA-gRNA architectures (encoding one gRNA) downstream of an EGFP transcript (Supplementary Fig. 10a, c), although all designed arrays achieved substantial fluorescent intensities, we observed a 3.6-fold less editing efficiency compared to hU6-gRNA architecture for single-site editing (Supplementary Fig. 10b, d), suggesting that, to achieve high MBE efficiency, the hCtRNA-gRNA array should not be placed downstream of an mRNA transcript, for example, downstream of the gene editor. Taken together, these results establish a robust nCas9 based MBE strategy by using DAP arrays without any additional promoters to achieve highly efficient MBE in human cells.

### Large-scale MBE with DAP arrays

**Fig. 3:**
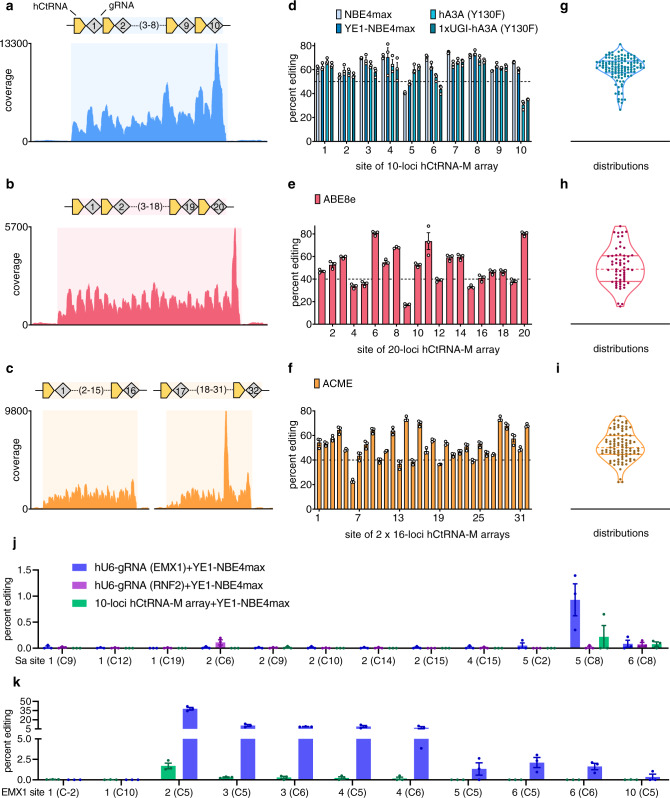
DAP strategy enables large-scale MBE with minimal off-target effect. **a**–**c** Schematic of RNA-seq results mapped onto the hCtRNA-gRNA multiplex arrays. **d** CBE multiplex editing of 10 loci using array shown in **a** with dash line indicating 50% editing. CBE architecture of each variant in **d** is shown in Supplementary Fig 12. **e** ABE8e multiplex editing of 20 loci using array shown in **b** with dash line indicating 40% editing. **f** 31-loci MBE using dual-deaminase base editor ACME and two 16-loci arrays in **c**, with dash line indicating 40% editing. gRNA 14 and gRNA 28 are identical and exhibit the same results in **f**. **g** Violin plot showing the value distribution of all replicates of **d** with solid lines representing quartiles, 57.4%, 67.3%, and dash line indicating median, 62.3%. **h** Violin plot showing the value distribution of all replicates in 20-loci MBE, with solid lines representing quartiles, 37.78%, 60.69%, and dash line indicating median, 48.68%. **i** Violin plot showing the distribution of all replicates of 31-loci MBE, with solid lines representing quartiles, 44.61%, 59.86%, and dash line indicating median, 50.12%. All tests were performed in HEK293T cells and analyzed using NGS. **j** Cas9-independent off-target editing evaluation of MBE at five Sa (SaCas9) sites (1,2,4,5,6) used in orthogonal R-loop assay^42^. **k** Cas9-dependent off-target editing evaluation of MBE at seven GUIDE-seq^44^ identified EMX1 off-target sites. Detailed protospacers, edited bases, amplicons, primers, and plasmid maps of the relevant figures are available in Supplementary Tables 1 and 5. Error bars represent mean ± s.e.m. from *n* = 3 replicates.

Next, to increase the scalability of MBE, we streamlined the hCtRNA-M array assembly method (Supplementary Fig. 11) and constructed 10-loci, 16-loci, and 20-loci hCtRNA-M arrays for MBE. We first performed RNA sequencing (RNA-seq) analysis on HEK293T cells expressing the 10-loci array, 20-loci array, and two pooled 16-loci arrays, respectively. The sequencing results revealed the transcription of the hCtRNA-M array and showed significant reads coverage of all hCtRNA and gRNAs in the array-located regions (Fig. 3a–c). We then performed large-scale MBEs by transfecting HEK293T cells with the hCtRNA-M arrays and various base editors (Method). For 10-loci MBE, we constructed several CBE variants based on the NBE4max architecture with different deaminases or UGI copy numbers (Supplementary Fig. 12). We observed efficient editing by NBE4max (averaging 63.7 ± 10.3%), hA3A (Y130F)^38^ (59.4 ± 11.1%), 1xUGI-hA3A (Y130F)^38^ (57.3 ± 10.3%), YE1-NBE4max^42^ (63.4 ± 6.9%), ABE8e^30^ (55.1 ± 11.7%) and ABE8e (V106W)^30^ (56.9 ± 12.9%) (Fig. 3d, g, Supplementary Figs. 12–16). We also performed dosage titration assays by using different amounts of the 10-loci hCtRNA-M array (0.75 ng to 225 ng). Substantial MBE editing efficiencies (averaging 10.8 ± 10.3%) could be observed when only 0.75 ng input of hCtRNA-M array was used. (Supplementary Fig. 14b, c). The previous study has shown that removing the UGI part of CBE can convert CBE into CGBE^43^, consistently, we observed 10-loci multiplex C-to-G editing using 1xUGI-hA3A (Y130F) (averaging 5.5 ± 9.1%) and 0xUGI-hA3A (Y130F) (averaging 16.9 ± 15.5%) (Supplementary Fig. 12, Supplementary Fig. 17). For 20-loci MBE, we were able to obtain high editing efficiency across all 20 sites, averaging 50.9 ± 16.6% by using ABE8e (Fig. 3e, h, Supplementary Fig. 18). We then developed a dual-deaminase base editor by fusing TadA-8e (V106W)−1xUGI-hA3A (Y130F) deamination module at N-terminal of nCas9 (D10A), hereafter referred to as ACME (A-to-G and C-to-T Multiplex Editor) (Supplementary Fig. 19a, b). By pooling two 16-loci arrays (one gRNA is identical in both arrays) together with ACME, we observed 51.3 ± 11.5% average editing of 31 different loci (Fig. 3f, i, Supplementary Fig. 19c). To evaluate the off-target activity of MBE, we performed both Cas9-independent and Cas9-dependent off-target DNA editing experiments in HEK293T cells^42,44^. In the orthogonal R-loop assay, we showed that hCtRNA-M array (the 10-loci array as shown in Fig. 3d encoding gRNAs targeting EMX1, RNF2, and other eight sites) with YE1-NBE4max exhibited comparable or less level of Cas9-independent off-target editing compared with hU6-gRNA (EMX1 or RNF2) with YE1-NBE4max (Fig. 3j). In the Cas9-dependent off-target assay, hCtRNA-M array with YE1-NBE4max displayed nearly undetectable off-target editing, while using hU6-gRNA with YE1-NBE4max showed significantly higher off-target editing (Fig. 3k). Together, these results demonstrate highly efficient and scalable MBE using the DAP strategy, without eliciting higher off-target editing.

### Multiplex prime editing with DAP arrays

**Fig. 4:**
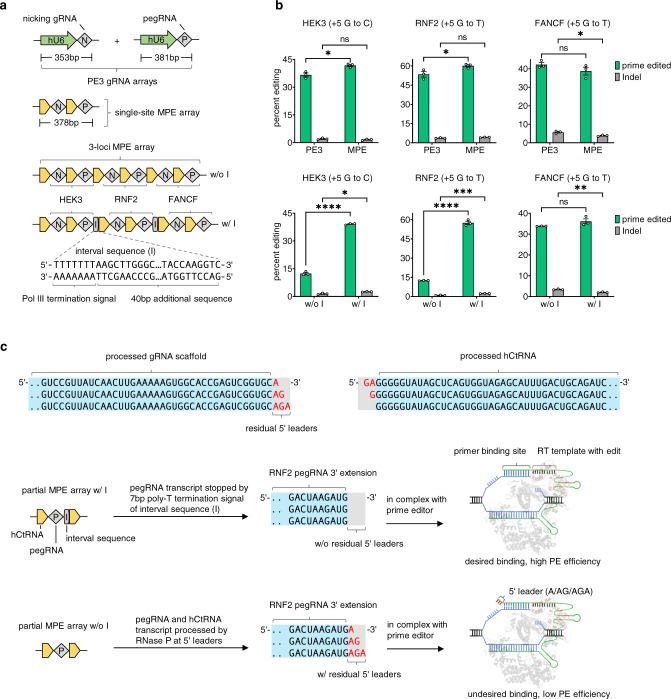
MPE with DAP arrays. **a** Schematic of MPE development. The size of PE3 gRNA arrays and single-site MPE array were compared using gRNA sequences designed for RNF2 (+5G to T). **b** Efficient single-site and 3-loci MPE. The w/o I and w/ I arrays were compared for 3-loci MPE. **c** Schematic illustration of the observed low 3-loci MPE efficiency (w/o I) in **b**. After the endogenous processing of the MPE array without interval sequence (w/o I), partial or complete 5′ leader sequences will remain at the 3′ end of pegRNA, which causes undesired primer binding and decreases the PE efficiencies. By adding the interval sequence (w/ I) at the 3′ end of pegRNA, the poly-T termination signal will isolate the original 3′ end of pegRNA from additional sequences, achieving desired binding and expected PE efficiencies. All tests were performed in HEK293T cells and analyzed using NGS. Detailed protospacers, edited bases, amplicons, primers, and plasmid maps of the relevant figures are available in Supplementary Tables 1 and 5. Error bars in **b** represent mean ± s.e.m. from *n* = 3 replicates (unpaired two-tailed *t*-test; ns not significant; *P < 0.05; **P < 0.01; ***P < 0.001; ****P < 0.0001). *P* = 0.0146 (prime edited value comparison between MPE and PE3 for HEK3 (+5G to C)). *P* = 0.0444 (prime edited value comparison between MPE and PE3 for RNF2 (+5G to T)). *P* = 0.0166 (Indel value comparison between MPE and PE3 for FANCF (+5G to T)). *P* = 1.87E-06 (prime edited value comparison between w/o I and w/ I for HEK3 (+5G to C)). *P* = 0.0113 (Indel value comparion between w/o I and w/ I for HEK3 (+5G to C)). *P* = 4.44E−06 (prime edited value comparison between w/o I and w/ I for RNF2 (+5G to T)). *P* = 0.0006 (Indel value comparison between w/o I and w/ I for RNF2 (+5G to T)). *P* = 0.002 (Indel value comparison between w/o I and w/ I for FANCF (+5G to T)).

Because BE only provides limited types of nucleotide substitutions and cannot be used for targeted deletion or insertion^6^. To expand the scope of multiplex precision gene editing, we sought to develop MPE based on the PE3 system^3^. Since two gRNAs (a pegRNA and a nicking gRNA) are necessary to achieve high PE efficiency at a single site and our DAP strategy could be beneficial in reducing the delivery size of the PE3 system. First, we assembled the nicking gRNA upstream of the pegRNA using the hCtRNA-M architecture (Fig. 4a). For single-site prime editing, we achieved similar or more efficient desired editing and similar or fewer insertions and deletion (Indel) rates compare to PE3 at sites including HEK3, RNF2, and FANCF (Fig. 4b). Next, to enable simultaneous editing of all three loci, we assembled six gRNAs on one hCtRNA-M array (Fig. 4a). Unexpectedly, only one site (FANCF), of which the gRNAs pair were located at the most downstream of the hCtRNA-M array, exhibited substantial editing efficiency (averaging 33.8 ± 0.1%), while the other two sites, HEK3 and RNF2 showed much lower efficiencies (Fig. 4b).

As the previous study has shown that a portion of the 5′ leader sequence can remain at the 3′ of gRNA after tRNA endogenous processing^31^, which is consistent with our RNA-seq data that the coverage gaps at the 5′ leader region were not evenly distributed (Supplementary Fig. 20). We thus inferred that the residual 5′ leader sequence at 3′ extension of pegRNA might influence the binding of the RNA-DNA complex to the M-MLV reverse transcriptase of the prime editor, thereby resulting in low PE efficiencies (Fig. 4c). To further improve the 3-loci MPE efficiency at HEK3 and RNF2 sites, we inserted an interval sequence (containing 7-bp poly-T termination signal) between 3′ extension and 5′ leader on the hCtRNA-M array, referred to as w/I (with interval sequence), as compared to previous array w/o I (without interval sequence) (Fig. 4a, c). Strikingly, we observed MPE efficiencies increased from 12.4 ± 1.0% (w/o I) to 39.1 ± 0.4% (w/I) at HEK3 site, and 12.5 ± 0.2% (w/o I) to 57.5 ± 2.3% (w/I) at RNF2 site. The multiplex editing efficiency at the FANCF site also slightly increased from 33.8 ± 0.1% (w/o I) to 36.1 ± 2.1% (Fig. 4b). Thus, our 3-loci MPE using engineered hCtRNA-M (w/I) achieved similar or higher editing efficiencies than single-site MPE or PE3 (Fig. 4b). These results establish efficient and scalable MPE with further engineered DAP arrays containing interval sequences.

### MBE of disease relevant-loci and AAV delivery of MBE cassette

**Fig. 5:**
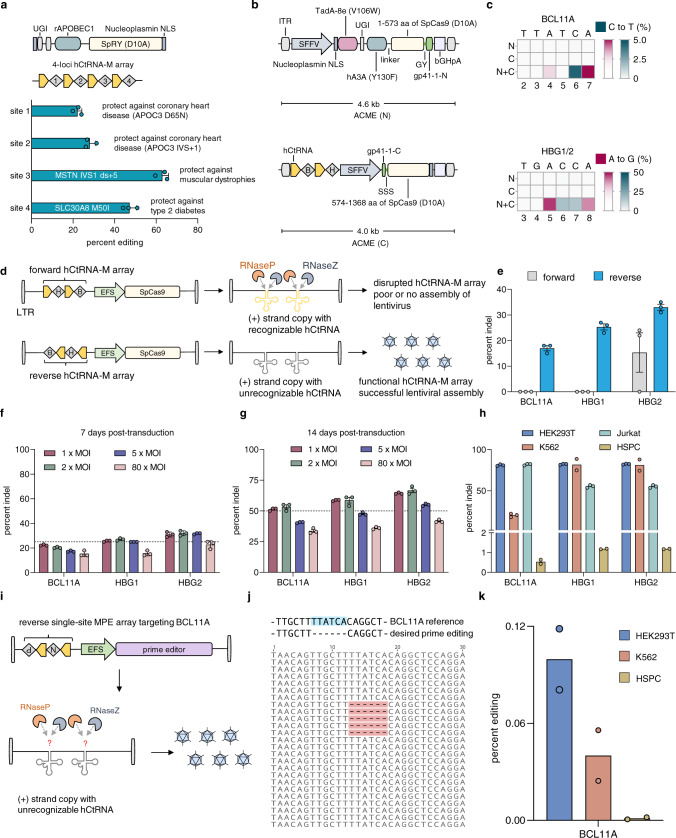
MBE of polygenic disease-relevant loci, adapting MBE and MPE to viral delivery for collective installation of disease-suppressing mutations. **a** MBE of polygenic disease-relevant loci in HEK293T cells. **b** Dual AAV vectors encoding optimized split ACME and hCtRNA-M array. SFFV, spleen focus-forming virus promoter; B and H represent gRNAs of BCL11A target 2 and HBG1/2 protospacer, respectively (Supplementary Fig 22); GY, extein amino acid residues Gly (G) Tyr (Y); SSS, extein amino acid residues Ser (S) Ser (S) Ser (S); gp41-1-N/C, N- or C-terminal gp41-1 trans-splicing intein; ITR, inverted terminal repeat. **c** Heat maps showing MBE in HEK293T cells using dual AAV vectors depicted in **b**, packaged as AAV1 pseudotype. Values represent the mean of three biological replicates. **d** Schematic of lentiviral Cas9 constructs with the hCtRNA-M cassette in forward and reverse orientations. LTR, long terminal repeat; B and H represent gRNAs of BCL11A target 1 and HBG1/2 protospacer (Supplementary Fig 22); EFS, elongation factor-1α short promoter. **e** Comparing multiplex editing between the two lentiviral constructs depicted in **d**, showing (+) strand RNA copy with a recognizable forward hCtRNA-M array is susceptible to RNase P and Z. **f**, **g** Optimization of lentiviral transduction for multiplex Cas9 nuclease editing in HEK293T cells (Methods). Lentiviruses containing reverse hCtRNA-M array depicted in **d** were used. **h** Multiplex Cas9 nuclease editing of BCL11A and HBG1/2 in HEK293T, K562, Jurkat cells, and primary human CD34 + HSPC, followed by 27 days of puromycin selection for three cell lines and 20 days for HSPC. **i** Schematic of lentiviral MPE constructs non-susceptible to endogenous tRNA processing. N, nicking gRNA; P, pegRNA. **j** The representative on-target MPE editing reads with desired deletion editing highlighted in red. **k** MPE at BCL11A site in HEK293T, K562 cells and HSPC using lentivirus encoding PE2 and a reverse single-site MPE array assembling pegRNA and nicking gRNA, followed by 20 days of puromycin selection. Detailed protospacers, edited bases, amplicons, primers, and plasmid maps of the relevant figures are available in Supplementary Tables 1 and 5. Error bars represent mean ± s.e.m. from *n* = 3 replicates.

To explore the therapeutic potential for our MBE strategy, we next used MBE to simultaneously install multiple protective genetic variants against polygenic diseases in human cells. We designed a 4-loci hCtRNA-M array and a CBE variant using near-PAMless Cas9^26^ (NBE4max-SpRY) and transfected them in HEK293T cells for three days before NGS analysis. At the relevant loci, we observed on-target C-to-T conversions implicated in protecting individuals against coronary heart disease with 22.14 ± 2.1% and 28.1 ± 2.8% efficiencies, type 2 diabetes with 47.2 ± 3.4% efficiency, muscular dystrophies with 63.0 ± 3.0% efficiency (Fig. 5a, Supplementary Fig. 21).

Previous studies have shown that the editing of either BCL11A (mutating GATA1 binding motif) or HBG1/2 loci (mutating BCL11A binding motif) can induce the upregulation of fetal hemoglobin (HbF) and thus could be a promising therapeutic strategy for treating sickle cell disease (SCD) and β-thalassemia (Supplementary Fig. 22)^45,46^. To demonstrate that multiple disease-suppressing edits could be installed simultaneously, we designed a 3-loci hCtRNA-M array encoding two gRNAs targeting BCL11A and HBG1/2 (the protospacers of HBG1 and HBG2 are identical). Tested by various base editors, we observed up to 42.0 ± 1.0% (BCL11A) and 58.3 ± 2.5% (HBG1/2) editing efficiencies. Assembling three copies of the 3-loci array increased the editing efficiency slightly to 44.3 ± 1.5% (BCL11A) and 61.3 ± 2.1% (HBG1/2) (Supplementary Fig. 23a, Supplementary Fig. 24).

Further, we split the dual-deaminase base editor (ACME) into two AAV vectors using a smaller trans-splicing intein gp41-1 than previously used Npu intein^47,48^ (Fig. 5b). To improve the intein splicing efficiencies, we placed the native extein sequence of gp41-1 flanking the split joint to enhance the intein trans-splicing process (Supplementary Fig. 25). We used only two Nucleplasmin NLSs rather than four to facilitate the full exposure of the gp41-1-N and gp41-1-C for trans-splicing reactions, which led to 1.2-fold higher editing efficiencies (Supplementary Fig. 23d, e). In addition, an SSFV Pol II promoter^49^ (instead of the CMV promoter) improved the ACME efficiency by 1.2-fold approximately (Supplementary Fig. 23f, g). Together, the plasmid-transfection of engineered vectors achieved efficient MBE efficiencies up to 56.3 ± 1.5% at the BCL11A site and 73.7 ± 1.5% at HBG1/2 in HEK293T cells (Fig. 5b, Supplementary Fig. 23g).

We then packaged ACME and 3-loci hCtRNA array into 4 pseudotypes of AAV vectors, including AAV1, AAV2, AAV6, and AAV-DJ^50^ (Supplementary Fig. 23h). We performed transductions using prepared AAVs in HEK293T cells and observed that AAV1 achieved relatively higher efficiencies than the rest of AAVs, both in the MBE editing (up to 24.7 ± 2.3%) and the expression of a reporter GFP (Supplementary Fig. 23i, j). Further, we optimized the transduction conditions by transducing 5-fold fewer cells (from 7500 to 1500 cells per well) with dual AAV1 vectors and sequenced the targeted loci by NGS after 7 days, up to 41.0 ± 2.8% MBE efficiency was observed (Fig. 5c).

### Lentiviral delivery of MPE cassette

Next, we sought to package MPE cassette within a single lentivirus, although the size of the current single-site MPE cassette (including the prime editor, 2 gRNAs, and others, ~10 kb) has already reached the packaging limit (9−10 kb) of the lentivirus vector. To test if the hCtRNA-M array could be adapted to lentiviral delivery, we start with packaging the Cas9 nuclease via single lentiviral delivery. We replaced the hU6-gRNA cassette of the lentiCRISPR v2 vector^51^ (containing a Cas9 nuclease) with four different hCtRNA-M array designs encoding two gRNAs targeting HBG1/2 and BCL11A sites (Supplementary Fig. 23b, Supplementary Fig 22). The four designs include different gRNA orders on either a forward hCtRNA-M array (same direction as the EFS RNA Pol II promoter) or a reverse array on the lentiCRISPR v2 vector. We then transfected HEK293T cells with four plasmids and found that only by placing the gRNA of BCL11A downstream the hCtRNA-M array can enable substantial editing efficiency (more than 20% at each site), regardless of array directions (Supplementary Fig. 23c), suggesting a strategy to improve multiplex editing by permuting the gRNA orders on the hCtRNA-M array.

We then packaged lentiviruses using the two plasmids showing efficient BCL11A editing through a similar process as for AAV packaging (Method, Supplementary Fig. 23h), followed by transduction of HEK293T cells and puromycin selection for 6 days. Only the reverse hCtRNA-M array enabled effective lentiviral transduction and multiplex Cas9 nuclease editing (17.0 ± 1.7% at BCL11A; 25.3 ± 2.1% at HBG1; 33.0 ± 2.0% at HBG2), which might be because the endogenous RNase P/Z could recognize the (+) strand placed forward hCtRNA-M array and thus disrupt the lentiviral functions (Fig. 5d, e, Supplementary Fig. 26). Extending the puro-selection to 2 weeks with optimized transduction conditions, we observed more than 50% editing efficiency at all targeted loci (Fig. 5f, g). We further demonstrated multiplex Cas9 nuclease-editing using lentiviruses in four different types of human cells, including HEK293T (up to 82.5 ± 0.6% efficiency), K562 (81.7 ± 9.9%), Jurkat (81.9 ± 1.0%), and cord-blood derived hematopoietic stem and progenitor cells (HSPC) (1.2%), with 3 or 4 week- puro-selection (Fig. 5h). These results suggest using a reverse hCtRNA-M array for effective lentiviral delivery of multiplex gene cassette.

To enable lentiviral delivery for MPE, we designed five single-site MPE arrays (Method) for installing disease-suppressing deletions at BCL11A (deletion of GATA1 binding motif TTATCA) or HBG1/2 site (deletion of BCL11A binding motif TGACCA). We transfected HEK293T cells with each designed array and prime editor (pCMV-PE2) for three days before sequencing analysis. However, only one out of the five single-site MPE arrays exhibited observable deletion efficiency, reaching 10% of a precise 6-bp deletion at the BCL11A site (Supplementary Fig. 27), which is most likely due to the limited editing efficiency of the PE3 system, varying from cell types and genomic loci.

We then chose the effective BCL11A single-site MPE array and constructed a single lentiviral vector (based on lentiCRISPR v2) encoding both PE protein and the reversed BCL11A array to delete a 6-bp GATA1 binding motif in the BCL11A gene (Fig. 5i). We transduced HEK293T, K562, and HSPC cells followed by 20 days of puromycin selection, then sequenced the targeted region and calculated the desired PE efficiency. Using more than 100,000 aligned reads for each sample, we observed the precise 6-bp deletion of the GATA1 binding motif from both HEK293T and K562 samples, albeit low efficiency was achieved. These results demonstrate the delivery of the MPE systems to human cells using a single lentiviral vector (Fig. 5j, k, Supplementary Fig. 28).

## Discussion

Here, we develop a DAP strategy for MBE and MPE in human cells, via an engineered 75-nt hCtRNA, to express the hCtRNA-gRNA multiplex array and direct the release of individual gRNAs. We streamline the assembly of the DAP array and enable scalable multiplex editing of up to 31-loci MBE and up to 3-loci MPE. In addition, MBE with DAP array substantially minimizes the Cas9-dependent off-target DNA editing and did not elevate the Cas9-independent deamination. The minimized Cas9-dependent off-target activity of MBE with DAP arrays might be because the individual gRNA concentrations released by DAP arrays could be efficient for the on-target editing but not enough for off-target editing^52^. Of note, to achieve efficient MPE when targeting two or more sites, a poly-T containing interval sequence should be used in the DAP array. We demonstrate that our MBE or MPE systems are compatible with AAV and lentiviral delivery for simultaneous editing of multiple disease-relevant loci. Previous multiplex strategies, however, have been using upstream promoters such as 249-bp hU6^53^ (Supplementary Fig. 29) and 1733-bp CAG^54^ to drive the multiplex array or using heterologous expression of Cys4 RNase to facilitate the processing of gRNA array that can cause severe cytotoxicity^55^, these approaches also require larger packaging sizes that inevitably limit the in vivo applications.

The repetitive sequences in the hCtRNA-gRNA array could cause mutations in the array sequences during cloning. To address this limitation, based on our findings in 5′ leader engineering (Fig. 2b, c, Supplementary Fig. 2), we suggest using different tRNA in the multiplex array when sequence repeating must be minimized. For example, to construct a 2-loci multiplex array in lentivirus vector, using two different tRNAs, e.g., hCtRNA and hGtRNA (hGly GCC tRNA with 6 nt 5′ leader sequence, Supplementary Fig. 2), to drive and process each gRNA, respectively. Although most of the multiplex editing with DAP strategy is efficient, insufficient transcription of gRNAs on DAP arrays in some cases (Supplementary Fig 23b, c) can limit the editing efficiencies. Strategies such as permutation of the gRNA orders on the DAP array (Supplementary Fig 23b, c) can be further developed to improve the transcription abilities of the DAP array.

The application of MBE and MPE would enable complex biological research and sophisticated therapeutic modalities^56^. We anticipate that combining DAP strategy with more emerging genome engineering tools^57–59^, CRISPR screening methods^60–62^, and delivery technologies^63,64^ would continue providing promising avenues for basic biology, crop engineering^65^, and therapeutics.

## Methods

### Molecular cloning

DNA amplifications were performed by PCR using 2 × Phanta Max Master Mix (Dye Plus, Vazyme). Vectors were linearized mainly by PCR and alternatively by restriction digestions. DNA of interest larger than 5 kb was split into smaller pieces for amplification. Typically, a 20 µl PCR reaction system with 60 °C annealing temperature and 25-cycle amplification was programmed for amplifying 0.5–5 kb DNA; 35-cycle amplification was set for DNA < 0.5 kb. A 51–55 °C annealing temperature was recommended for preparing 0.1–0.3 kb DNA fragments of the hCtRNA-M array (Supplementary Fig. 11). Amplified DNA was purified by gel extraction using QIAquick Gel Extraction Kit (Qiagen) or FastPure Gel DNA Extraction Mini Kit (Vazyme), DNA was run and cut in the small type well of 1% DNA agarose gel stained by UltraPure Ethidium Bromide (Thermo Fisher Scientific) to a final concentration of 0.5 μg/ml. All plasmids were designed on Benchling and constructed mainly by Golden Gate assembly (Assembly Wizard, Benchling), alternatively by Gibson assembly. Typically, a 10 µl Golden Gate assembly system containing purified DNA pieces, 1 µl 10 x T4 DNA ligase buffer (New England BioLabs), 0.5 µl T4 DNA ligase (200 U, New England BioLabs), and 0.5 µl BsaI-HFv2 (10 U, New England BioLabs) or Esp3I (5 U, Thermo Fisher Scientific) was cycled between 37 °C and 16 °C for 5 min at each temperature for 15 cycles then subjected to a 60 °C incubation for 5 min; a 5 µl or 10 µl Gibson assembly system (ClonExpress Ultra One Step Cloning Kit, Vazyme) containing purified DNA pieces and the enzymatic mix was incubated at 50 °C for 15 min. Transformations were performed using Stbl3 competent cells (10^8^−10^9^ CFU/μg DNA) prepared by Mix & Go! *E. coli* Transformation Kit (Zymo).

DNA oligonucleotides were obtained from Integrated DNA Technologies (IDT), the final concentration of each primer in a PCR reaction was 0.5 µM. Plasmids containing tRNA variants (Supplementary Tables 2 and 3) were initially constructed by ligation of annealed oligonucleotides (DNA sequence of tRNA) with other amplified DNA parts through Golden Gate assembly. Typically, a 10 µl annealing system containing 10 µM of each oligonucleotide, 1 µl 10 x T4 DNA ligase buffer (New England BioLabs) and 0.5 µl T4 Polynucleotide Kinase (5 U, New England BioLabs) was incubated at 37 °C for 30 min then 95 °C for 5 min followed by a −5 °C/min ramp down to 25 °C, the annealed oligonucleotides were diluted to a final concentration of 0.04–1 µM for Golden Gate assembly. Addgene plasmids BE4max (#112093), pCMV-ABE7.10 (#102919), LbABE8e (#138504), hA3A-BE3 (#131314), pCMV-PE2 (#132775), lentiCRISPR v2 (#52961), pAAV-EF1a-Flpo (#55637), CBE4max-SpRY (#139999), SiT-Cas12a (#128405), TRE3G-EGFP (#52343) were used directly or as PCR template. Npu and gp41-1 inteins were codon-optimized (GenSmart, GeneScript) for human cell expression and synthesized by gBlocks (IDT).

Plasmids were isolated using QIAprep Spin Miniprep Kit (Qiagen) or FastPure Plasmid Mini Kit (Vazyme) and eluted in the kit-provided elution buffer. Mini Spin column (Epoch Life Science) was alternatively used in all described miniprep and gel extraction kits. Constructs were verified by Sanger sequencing across all assembly junctions, the coding sequence of the deaminase-containing construct was fully confirmed. The annotated sequence of each key plasmid developed by this work is available in the shared Benchling links (Supplementary Table 1).

### Mammalian cell culture

HEK293T cells (ATCC CRL-3216) were cultured in Dulbecco’s modified Eagle’s medium (DMEM) plus GlutaMAX (Gibco) supplemented with 10% (v/v) fetal bovine serum (Gibco, qualified) and 1% (v/v) penicillin-streptomycin (Gibco). K562 (ATCC CCL-243) and Jurkat (ATCC TIB-152) cells were cultured in Roswell Park Memorial Institute (RPMI) 1640 medium plus GlutaMAX (Gibco) supplemented with 10% (v/v) fetal bovine serum (Gibco, qualified) and 1% (v/v) penicillin-streptomycin (Gibco). Human cord-blood-derived primary CD34+ hematopoietic stem and progenitor cells (HSPC) (70008.2, StemCell Technologies) were cultured in StemSpan SFEM II (StemCell Technologies) supplemented with 1 x StemSpan CD34 + expansion supplement (StemCell Technologies), 1 μM UM729 (StemCell Technologies) and 1% (v/v) penicillin-streptomycin (Gibco). Cells were authenticated by the supplier using STR (short tandem repeat) analysis. All described cells were grown at 37 °C in 5% CO2 incubators and passaged upon reaching 80% confluency. Cell culture media was tested for mycoplasma contamination every 2 months using the Myco-Blue Mycoplasma Detector (Vazyme) and all tests were negative throughout the experiments.

### Transfection

HEK293T cells were passaged every other day at a split ratio of 1:4, cells of low passage number (1−10, freshly thawed counted as 0) were counted by Countess II FL (Thermo Fisher Scientific) and plated at 0.75 × 10^4^ cells per 100 µl culture medium per well in 96-well poly-D-lysine coated plates (Corning) 16 h before transfections, the seeded plate was pre-incubated at room temperature for 15 min before placing into the incubator to reduce the edge effect and avoid unevenly seeded cells^66^. For each well on the plate, transfection plasmids and a constant 0.5 μl Lipofectamine 2000 (Thermo Fisher Scientific) were separately diluted in Opti-MEM I Reduced Serum Medium (Thermo Fisher Scientific) to 5 μl of each, then combined to a total of 10 μl and incubated for 5 min at room temperature before pipetted onto the supernatant. For MBE and MPE, typically, each well was transfected with 225 ng of base/prime editor plasmid and 75 ng of gRNA plasmid containing 1-5 gRNAs or 225 ng of gRNA plasmid containing more than 5 gRNAs, pooled-gRNA delivery was performed by combining equal mass of each gRNA plasmid to a total of 75 ng. For 31-loci MBE, each well was transfected with 250 ng of the ACME plasmid and 250 ng of gRNA plasmid containing two 125 ng 16-loci hCtRNA-M plasmids (Supplementary Table 1). For PE3, each well was transfected with 225 ng PE2 plasmid, 75 ng pegRNA plasmid, and 25 ng nicking gRNA plasmid. For intein-split BE, the mole value of each partial editor plasmid was kept the same as the 225 ng full-length editor plasmid. Plasmid based on lentiCRISPR v2 vector was transfected 500 ng per well. EGFP containing plasmid was transfected 225 ng per well. In orthogonal R-loop assays to test Cas9-independent off-target DNA editing, 200 ng of base editor plasmid and 100 ng of gRNA or DAP array plasmid, 200 ng of dSaCas9 and SaCas9 gRNA all-in-one plasmid were transfected into HEK293T cells. No filler plasmid was used in all transfections. Genomic DNA was extracted 72 h post-transfection for gene editing analysis. Flow cytometry analysis was performed 48 h post-transfection.

### Genomic DNA extraction

The medium of each well was gently aspirated (for HEK293T cells), or firstly centrifuged at 500 g for 3 min and then aspirated (for K562, Jurkat, and HSPC cells), followed by the addition of 50 µl/well freshly prepared lysis buffer (10 mM Tris-HCl, pH 7.5, 0.05% SDS, 25 µg ml−1 proteinase K (Thermo Fisher Scientific)) and incubated at 37 °C for strictly 1 h then heat-inactivated at 80 °C for 30 min. Genomic DNA lysate was immediately used or stored at 4 °C until use.

### Flow cytometry

6–16 h post-transfection of the construct expressing EGFP, the fluorescence of each well was verified and imaged using EVOS FLoid Imaging System (Thermo Fisher Scientific). 48 h post-transfection, the medium of each well was gently aspirated, followed by the addition of 100 µl/well TrypLE Express (Thermo Fisher Scientific) and incubated at 37 °C for 10 min, then diluted with 100 µl/well culture medium (1 % (v/v) FBS). Flow cytometry was performed using FACSCanto II Flow Cytometer (BD Biosciences) and analyzed using FlowJo 10.7.1 (FlowJo, LLC). Cells were gated by forward versus side scatter (FSC vs. SSC) plot to identify cell population and exclude debris, forward scatter height versus forward scatter area (FSC-H vs. FSC-A) plot for doublet exclusion, and FSC-H or histogram vs. FITC-A plot to reflect EGFP signal. All represented samples were assayed with three biological replicates. Data is representative of at least 5,000 gated events per condition.

### Lentivirus and AAV production

Low passage HEK293T cells were seeded at 5 × 10^6^ cells per 10 ml culture medium per 10-cm cell culture dish 16 h before transfection. For lentivirus production in each dish, 5 μg of vector plasmid containing the construct of interest, 2.5 μg of pMD2.G plasmid (Addgene, #12259), and 4.5 μg of psPAX2 plasmid (Addgene, #12260) were added to 260 μl of serum-free DMEM in a 50-ml tube, followed by addition of 78 μl transfection reagent PEI Max (1 mg/ml, PH = 7.1, Polysciences), vortexed and then incubated at room temperature for 10 min. After incubation, the transfection mixture was diluted with 10 ml of culture medium and used to replace the old medium of the 10-cm dish. 48 h post-transfection, 10 ml of supernatant was collected in a 15-ml tube and centrifuged at 3200 *g* for 5 min at room temperature to remove the cell debris, then clarified through a 0.45 μm PVDF filter (Millipore) and concentrated using PEG virus precipitation kit (Biovision) with an optimized protocol. Briefly, 2.5 ml of PEG solution was added to the 10 ml supernatant, inverted evenly, and refrigerated at 4 °C for 24 h, then centrifuged at 3200 *g* and 4 °C for 30 min, followed by several rounds of aspiration and centrifugation to entirely remove the supernatant from the precipitated white pellet at the bottom of the tube. The pellet was then suspended in 80 μl of virus resuspension solution. The overall process for AAV production was the same as did for lentivirus except for the plasmid used (Supplementary Fig. 23h). For each dish, 3 μg of vector plasmid, 5 μg of pHelper plasmid (Cell Biolabs), and 4 μg of AAV-Rep-Cap plasmid (AAV2 (Addgene, #104963), AAV1 (Addgene, #112862), AAV-DJ (Cell Biolabs)) were transfected. AAV6 pseudotype was prepared using 3 μg of vector plasmid and 9 μg of pDGM6 (Addgene #110660). The freshly prepared lentivirus or AAV were immediately used for transductions. Lentiviral titers were determined by transducing cells with different volumes of lentivirus (0–20 μl) and counting the number of surviving cells after a complete selection. AAV was titered by real-time quantitative PCR, droplet digital PCR (ddPCR), or flow cytometry analysis of the EGFP encoded in the vector.

### Transductions

Low passage cells were seeded at 500-1 × 10^5^ cells per 100 μl culture medium per well in 96-well poly-D-lysine coated plate (Corning) and pre-incubated at room temperature for 15 min, followed by addition of freshly prepared lentivirus or AAV, then placed into the incubator. To optimize the transduction efficiency, cells were transduced with different multiplicity of infection (MOI). For example in Fig. 5f, g, 1 x MOI, 2 x MOI, 5 x MOI and 80 x MOI represent 20 μl/well lentiviruses transducing 4 × 10^4^, 2 × 10^4^, 0.75 × 10^4^, 500 HEK293T cells per well, respectively. The optimal lentiviral transduction conditions were 2 × 10^4^ cells/well for HEK293T cells, 4 × 10^4^ cells/well for K562, Jurkat cells, and 1 × 10^5^ cells/well for HSPC cells, all transduced with 20 μl/well of freshly prepared lentivirus. 24 h after lentiviral transduction, cells of each well were dissociated and centrifuged at 500 *g* for 3 min to remove the supernatant, then resuspended in 500 μl of culture medium supplemented with 1 μg/ml puromycin (Thermo Fisher Scientific) and replated in a 24-well cell culture plate to initiate puromycin selections. The duration of puromycin selection was 1–4 weeks, as indicated explicitly in the figures and legends, during which the transduced cells were passaged every other day starting on day 2 after replating. Genomic DNA extractions were performed after the designated time course. For AAV transduction, HEK293T cells were seeded at 1500 cells per 100 μl culture medium per well in the 96-well plate, pre-incubated at room temperature for 15 min, added with 15 μl N-terminal and 15 μl C-terminal ACME vectors (approximately 10^8^ genome copies of each AAV vector), and placed into the incubator. On day 6 after AAV transduction, each transduced well was supplemented with a 100 μl fresh culture medium. Genomic DNA extractions were performed on day 7 post-transduction. To evaluate the AAV transduction efficiency, 2 × 10^4^ HEK293T cells per well were transduced by AAV encoding EGFP and analyzed by flow cytometry 48 h post-transduction.

### Targeted amplicon sequencing and data analysis

The genomic region flanking each targeted locus was amplified, purified, quantified, and sent for Sanger sequencing (premix, GENEWIZ) or next-generation sequencing (NGS) (Amplicon-EZ, GENEWIZ). Primers and amplicon sequences are available in the shared Benchling link (Supplementary Table 5). Partial Illumina adapters provided by Amplicon-EZ were added to the 5′ end of each forward and reverse primer. Typically, a 10 µl PCR reaction was performed with 0.5 µM of each forward and reverse primer, 1 µl genomic DNA extract, and 5 µl Phanta Max Master Mix, setting 60 °C annealing temperature and 35-cycle amplification. All primer pairs were able to amplify the desired fragments and pre-verified by DNA electrophoresis in a 1% agarose gel. PCR products were purified using QIAquick PCR Purification Kit (Qiagen) or FastPure Gel DNA Extraction Mini Kit (Vazyme), Mini Spin column (Epoch Life Science). Specifically, for Sanger sequencing, each amplicon was eluted in 20 µl ultrapure water (Millipore) and quantified by NanoDrop One (Thermo Fisher Scientific), the sequencing premix (15 µl) was prepared by adding 1 µl eluted DNA (10-20 ng) and 2.5 µl 10 µM sequencing primer into 11.5 µl ultrapure water; for NGS, multiple different amplicons were pooled together, then purified and eluted in 30 µl kit-provided elution buffer, quantified firstly by NanoDrop One (Thermo Fisher Scientific) to dilute the DNA concentration until 60-80 ng/µl, then using Qubit dsDNA HS Assay Kit (Thermo Fisher Scientific) to prepare approximately 500 ng amplicon in 25 µl kit-provided elution buffer for Amplicon-EZ. Sanger sequencing results (.ab1 files) were analyzed using EditR (http://baseeditr.com/) and ICE (https://ice.synthego.com/) for BE and Indel readouts, respectively. NGS results (FASTQ files) were analyzed using CRISPResso2 (http://crispresso2.pinellolab.org/) in batch mode, run by website or docker containerization system (https://www.docker.com/). BE result and deletion frequency of each DNA base were assessed using ‘Quantification_window_nucleotide_percentage_table.’ Indel frequencies of PE or Cas9 nuclease editing were calculated as the percentage of (sum of all ‘Modified’ reads – the sum of all ‘Only Substitutions’ reads)/(‘Reads_aligned_all_amplicons’), using the CRISPResso2 output table ‘CRISPResso_quantification_of_editing_frequency.txt.’ For deletion edit using MPE cassette, HDR mode was run in CRISPResso2 analysis with ‘discard_indel_reads’ on, the editing efficiencies were calculated as the percentage of (‘Reads_aligned_HDR’) /(‘Reads_aligned_all_amplicons’). Representative NGS reads surrounding the on-target MPE deletion editing site were visualized using Geneious Prime 2021.1. Specifically, the paired-ends FASTQ files were imported and merged using ‘Merge Paired Reads’ and mapped to the amplicon sequence using ‘Map to Reference’. Data were visualized using GraphPad Prism 8.4.0, values of interest < 5% (EditR results) were excluded.

### RNA seq and analyses

Plasmids containing hCtRNA-M arrays were transfected in low passage HEK293T cells using PEI Max (1 mg/ml, PH = 7.1, Polysciences). Specifically, HEK293T cells were seeded at 5 × 10^6^ cells per 10 ml culture medium per 10-cm cell culture dish 16 h before transfection, 26 μg of 10-loci hCtRNA-M array plasmid or 13 μg each of the two 16-loci hCtRNA-M array plasmids were added to 260 μl of serum-free DMEM in a 50-ml tube, followed by addition of 78 μl transfection reagent PEI Max, vortexed and then incubated at room temperature for 10 min. After incubation, the transfection mixture was diluted with 10 ml of culture medium and used to replace the old medium of the 10-cm dish. 48 h post-transfection, total RNA was harvested from cells using E.Z.N.A. Total RNA Kit I (Omega Bio-tek) and quantified using NanoDrop One (Thermo Fisher Scientific). RNA samples (2 μg each, >50 ng/μl) were submitted to the Cancer Genomics Center at The University of Texas Health Science Center at Houston. Total RNA was quality-checked using Agilent RNA 6000 Pico kit by Agilent Bioanalyzer 2100 (Agilent Technologies). RNA with an integrity number greater than 7 was used for library preparation. rRNA of 400 ng total RNA was depleted with NEBNext rRNA Depletion Kit (New England Biolabs). The RNAs with more than 70 nt were selected for libraries preparation. Then libraries were prepared with NEBNext Ultra II Directional RNA Library Prep Kit for Illumina (New England Biolabs) and NEBNext Multiplex Oligos for Illumina (New England Biolabs). The quality of the final libraries was examined using Agilent High Sensitive DNA Kit by Agilent Bioanalyzer 2100, and the library concentrations were determined by qPCR using the Collibri Library Quantification kit (Thermo Fisher Scientific). The libraries were pooled evenly and went for the paired-end 75-cycle sequencing on an Illumina NextSeq 550 System using High Output Kit v2.5 (Illumina). The RNA-seq data were processed and analyzed using Geneious Prime 2021.1. Briefly, FASTQ data of the same experiment were imported through automatic paired-end processing and grouped as a sequence list, then trimmed using BBDuk, assembled, and mapped to reference using plugin Bowtie2, all with the default setting. The data of coverage were exported and visualized using GraphPad Prism 8.4.0.

### RNA structure

RNA secondary structures were predicted using RNAstructure. (https://rna.urmc.rochester.edu/RNAstructureWeb/).

### Prime editing gRNA design

pegRNA and nicking gRNA were designed using PrimeDesign^67^.

(https://drugthatgene.pinellolab.partners.org/)

### Statistics and reproducibility

Values were reported as mean ± SEM or mean ± SD as indicated in the relevant figure legends or the descriptions. Groups were compared using the unpaired two-tailed *t*-test or multiple t-text with discovery determined using the two-stage linear step-up procedure of Benjamini, Krieger, and Yekutieli, with *Q* = 1%. The dashed line of the scatter dot plot represents the mean value of all dots plotted. The solid lines and dashed lines of the violin plot represent the quartiles and median. Biologically independent experiments reported here were performed by the same researcher using separate splits of the mammalian cell type used.

### Reporting summary

Further information on research design is available in the Nature Research Reporting Summary linked to this article.

## Supplementary information

Supplementary Information

Reporting Summary

## Data Availability

Targeted amplicon sequencing data and RNA-seq data have been deposited at the Sequence Read Archive: https://www.ncbi.nlm.nih.gov/sra/PRJNA745452. The tRNA relevant data in tRNA database (http://gtrnadb.ucsc.edu/) were used. Source data of each relevant figure are provided. Plasmids for hCtRNA-M array assembly, hCtRNA_FT and hCtRNA_VT, as well as other essential constructs developed by this work, are available on Addgene via https://www.addgene.org/Xue_Gao/. Source data are provided with this paper.

## Source data

Source Data

## Code availability

Customized code for analyses described in this study is available on Github (https://github.com/qichenyuan/Customized-CRISPResso2-Code)^68^.
