# Supplementary material for: Multiplex base- and prime-editing with drive-and-process CRISPR arrays
Source: Nat Commun. 2022 May 19;13:2771. doi: 10.1038/s41467-022-30514-1 (PMC9120480; doi:10.1038/s41467-022-30514-1)
Supplement: Supplementary file 1 — Supplementary Information [file 41467_2022_30514_MOESM1_ESM.pdf]

Supplementary Information

Multiplex base- and prime-editing with drive-and-process CRISPR arrays

Qichen Yuan[1], Xue Gao[1, 2, 3]*

1 Department of Chemical and Biomolecular Engineering, Rice University, Houston, TX, USA.

2 Department of Bioengineering, Rice University, Houston, TX, USA.

3 Department of Chemistry, Rice University, Houston, TX, USA.

* Corresponding Author: xue.gao@rice.edu (X.G.)

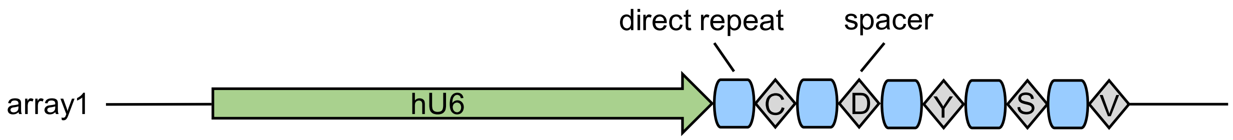

Supplementary Figure 1. 5-loci multiplex gRNA array for dLbCas12a MBE. hU6**,** human U6 promoter**.** C, CDKN2A; D, DNMT1; Y, DYRK1A; S, SITE3; V, VEGFA. 7-bp poly-T termination signal is not shown.

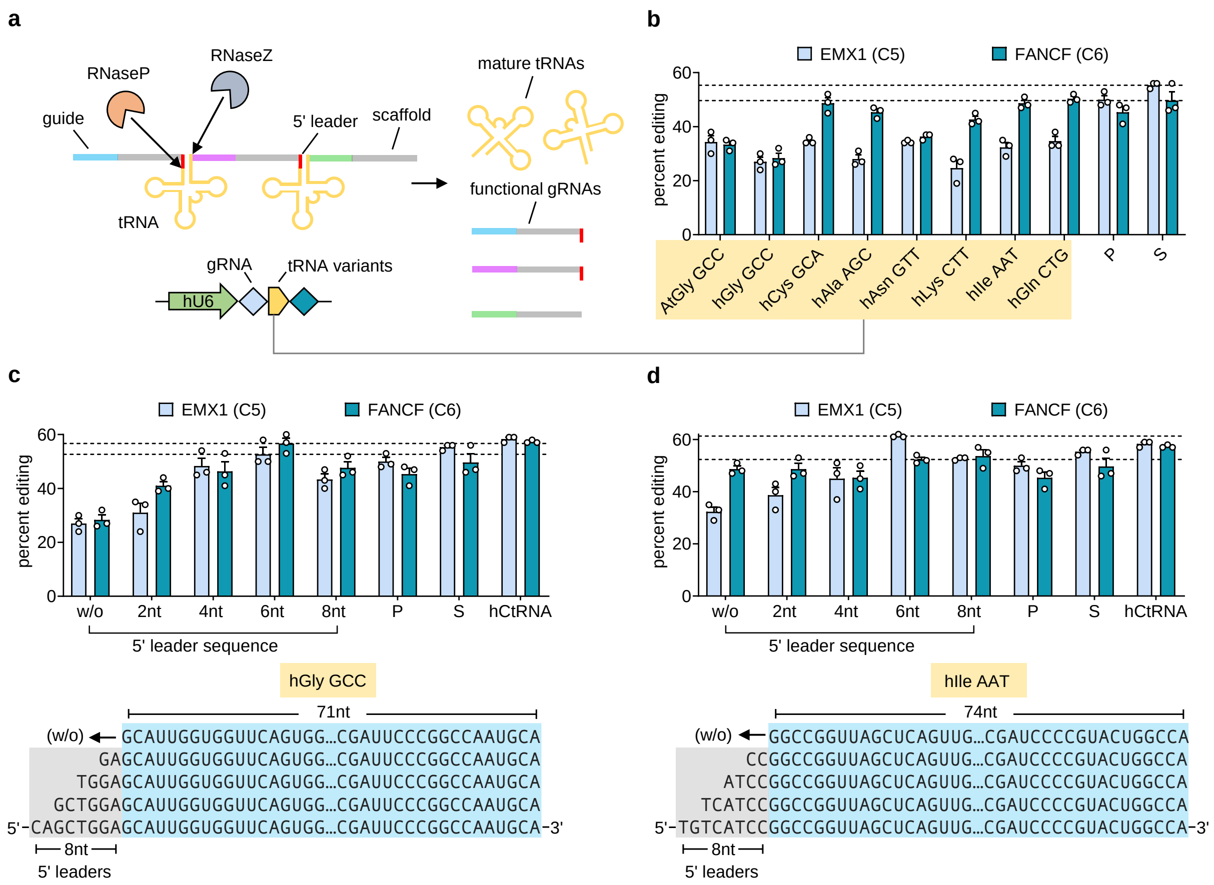

Supplementary Figure 2. MBE with mature or precursor tRNA variants in the gRNA-tRNA array. a, Schematic of the tRNA-gRNA processing mechanism and hU6-driven tRNA-gRNA architecture. hU6, human U6 Pol III promoter. **b**, Evaluation of 8 mature tRNA variants for 2-loci MBE. The abbreviation of mature tRNA variants, for example, AtGly GCC meaning a tRNA from Arabidopsis thaliana (At) with Glycine (Gly) isotype and GCC anticodon; hCys GCA is from human (h) with Cysteine (Cys) isotype and GCA anticodon. Dashed line showing mean editing efficiency using single gRNA (S). **c**,**d**, 5' leader engineering of hGly GCC and hIle AAT for efficient MBE. Dashed lines showing mean MBE efficiency with 6 nt 5' leaders. The w/o data (mature tRNA without 5' leaders) in **c** and **d** are from **b**. The data of P (pooled gRNAs), S (single gRNA), and hCtRNA (hCys GCA with 3 nt 5' leader sequence) in **b-d** are the same as in **Fig. 2c**. NBE4max (R33A+K34A) was used in all BE experiments. All tests were performed in HEK293T cells. Error bars represent mean ± s.e.m. from n = 3 replicates.

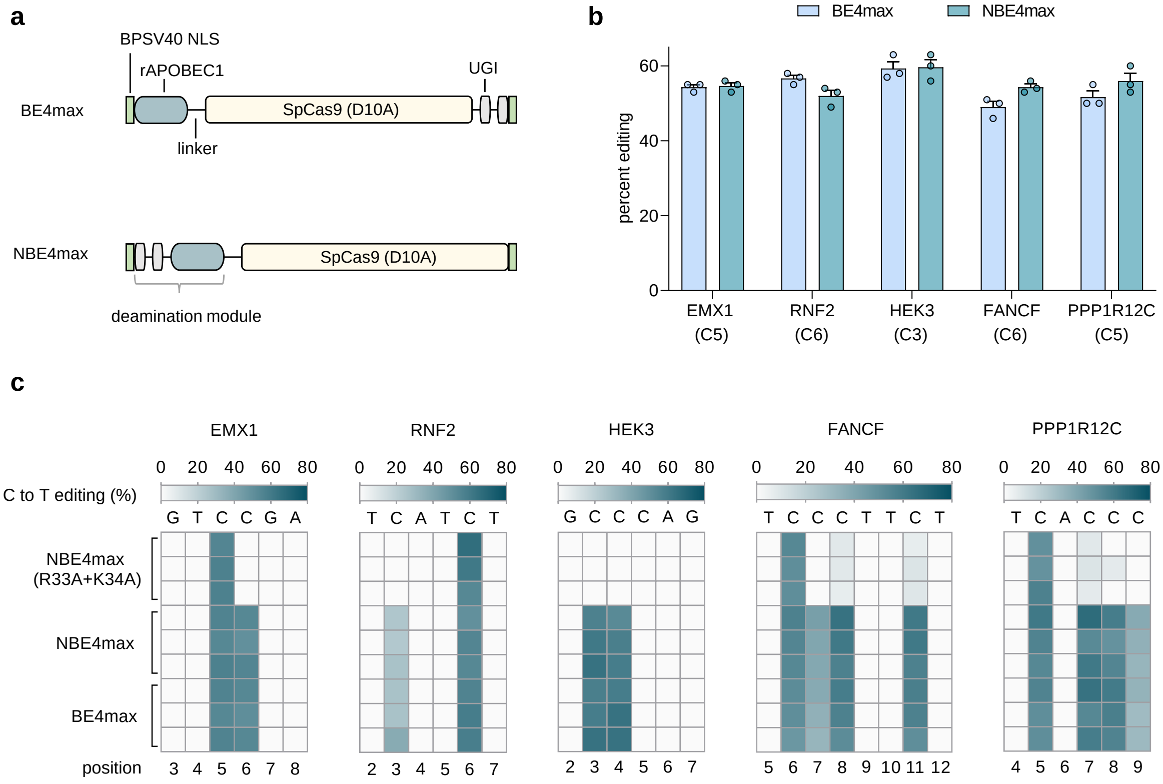

Supplementary Figure 3. NBE4max architecture with UGI part relocated at N-terminus of rAPOBEC1 cytidine deaminase. **a**, Schematic of BE4max and NBE4max. The 2 x UGI part of BE4max was moved to the N-terminus of rAPOBEC1 to construct NBE4max, fusing UGI and deaminase as a deamination module. **b**,**c**, Comparing base editing efficiencies of BE4max, NBE4max and NBE4max (R33A+K34A) at 5 genomic loci in HEK293T cells. Heat maps showing 3 independent replicates at each condition, counting 1 as the most PAM-distal position. Error bars represent mean ± s.e.m. from n = 3 replicates.

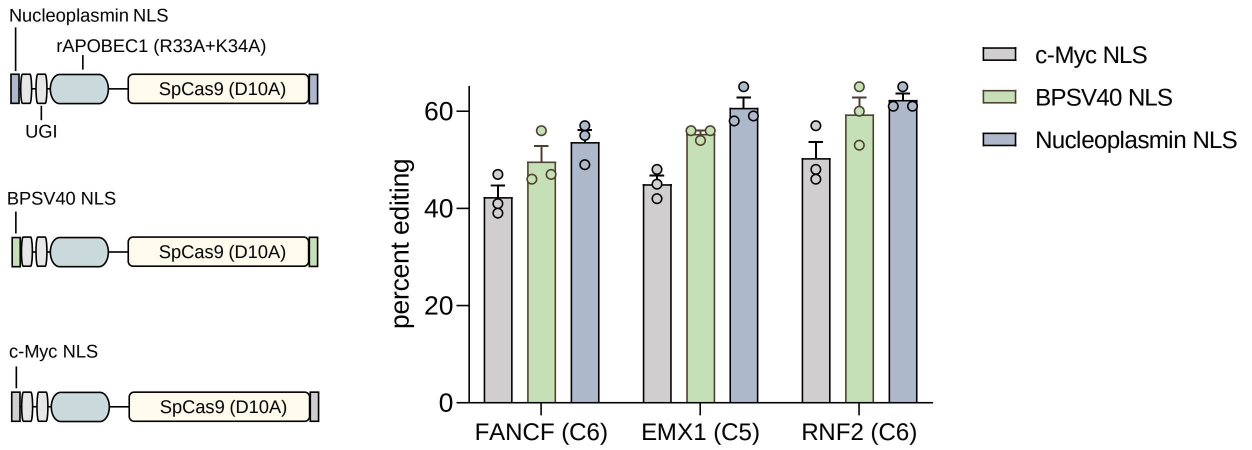

Supplementary Figure 4. Nucleoplasmin NLS enables higher base editing in HEK293T cells. Single-site base editing was performed by transfecting HEK293T cells with the hU6-driven gRNA cassette and NLS variant of NBE4max (R33A+K34A). Error bars represent mean ± s.e.m. from n = 3 replicates. Percent editing showing C-to-T conversion of the indicated DNA base within the protospacer, counting 1 as the most PAM-distal position.

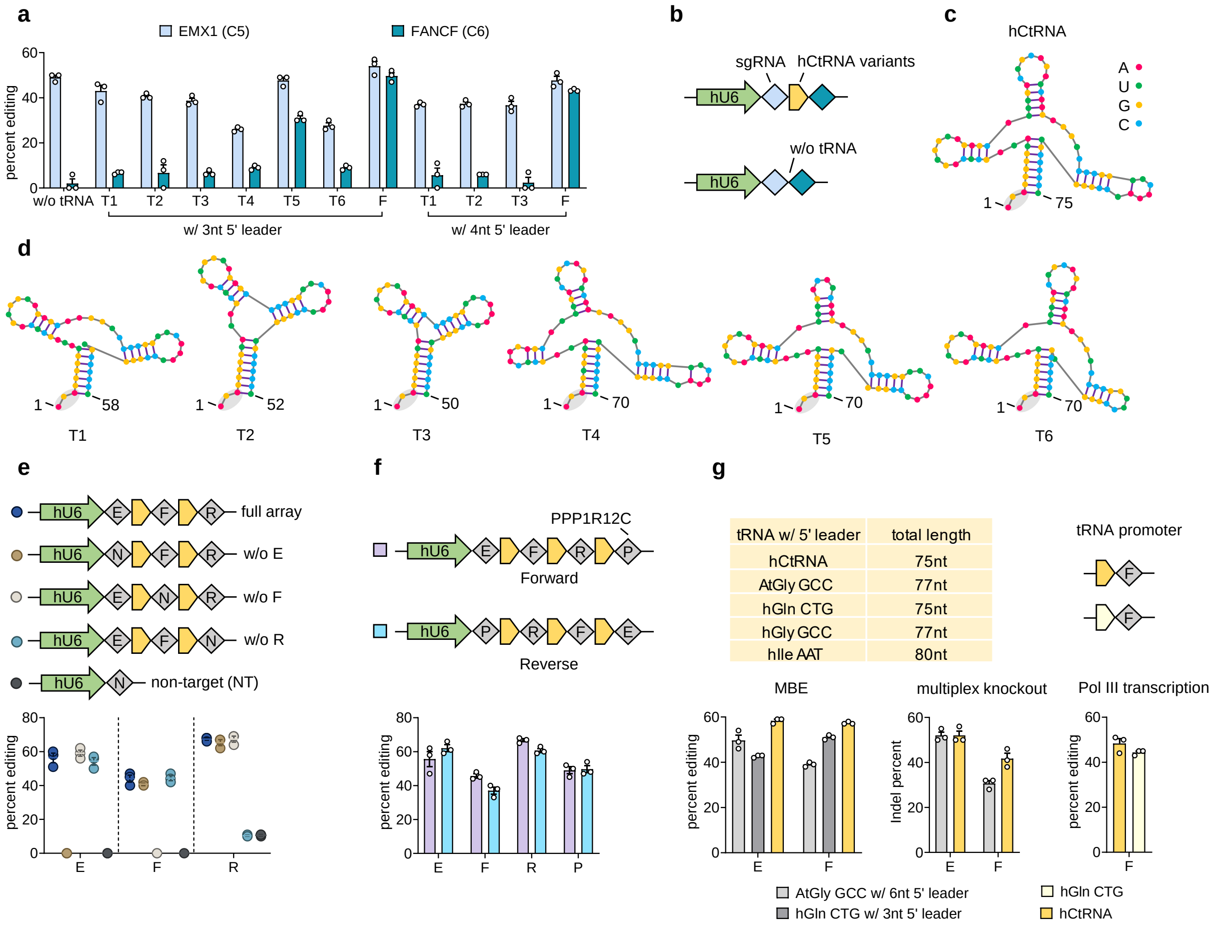

Supplementary Figure 5. MBE with truncated hCtRNA, MBE using different gRNA order, sequence-specific MBE, comparing hCtRNA with reported tRNAs. a, Truncated hCtRNA variants for MBE. F, full-length hCtRNA as shown in **c**; T1-T6 are hCtRNA truncations as shown in **d** (with 3 nt 5' leaders). **b**, Schematic of hU6-driven tRNA-gRNA architectures used in **a**. **c**,**d**, RNA secondary structures of hCtRNA and hCtRNA truncation variants. **e**, Targeting and non-targeting gRNA combinations on three-guide arrays showing sequence-specific MBE. E, EMX1 (C5); F, FANCF (C6); R, RNF2 (C6). **f**, reversing gRNA order of a four-guide array does not influence MBE performance. P, PPP1R12C (C5). **g**, Comparing hCtRNA with reported tRNA variants for MBE, the multiplex knockout with Cas9, and transcription of gRNA. NBE4max (R33A+K34A) was used in all BE experiments. All tests were performed in HEK293T cells. Error bars represent mean ± s.e.m. from n = 3 replicates.

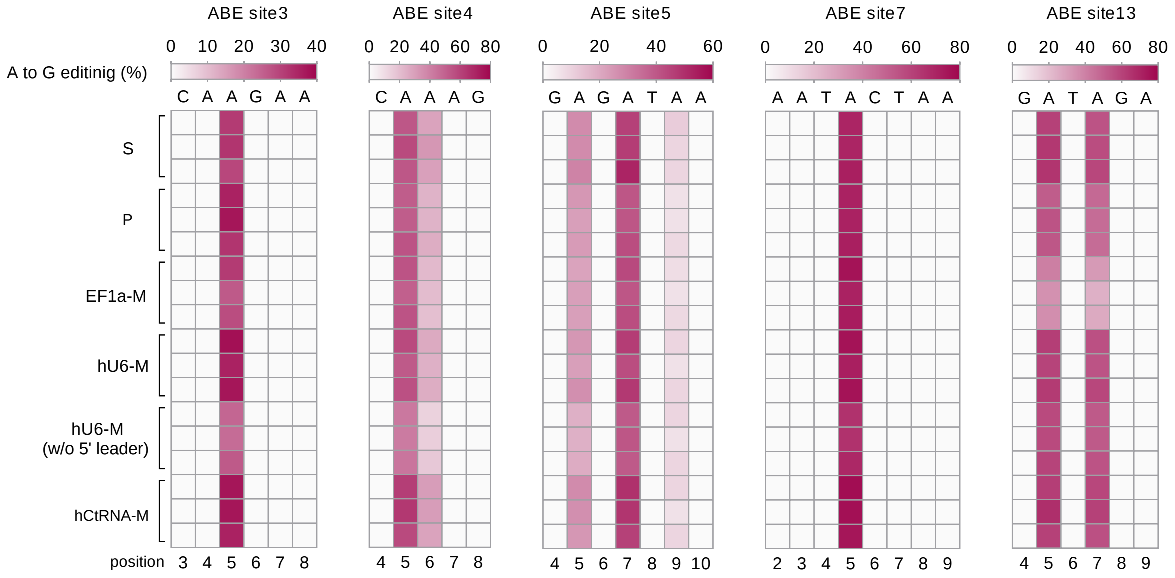

Supplementary Figure 6. The hCtRNA-M array enables efficient 5-loci MBE in HEK293T cells. Heat maps showing ABE 7.10 (F148A) multiplex editing at 5 genomic loci. 4 different architectures of gRNA array depicted in **Fig. 2g** were compared. Base editing efficiencies were analyzed by Sanger sequencing.

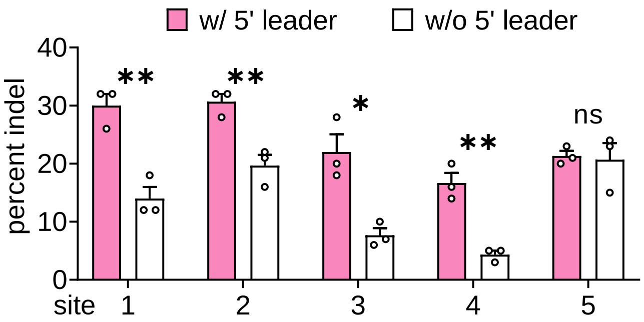

Supplementary Figure 7. Comparison of mature hCtRNA (w/o 5' leader) and hCtRNA (w/ 5' leader) for 5-loci multiplex knockout by Cas9 nuclease using hU6-M array architecture in HEK293T cells. Error bars represent mean ± s.e.m. from n = 3 replicates (unpaired two-tailed t-test; ns, not significant; *P < 0.05; **P < 0.01; ***P < 0.001; ****P < 0.0001). P = 0.004813 (site 1), 0.008563 (site 2), 0.012006 (site 3), 0.002824 (site 4).

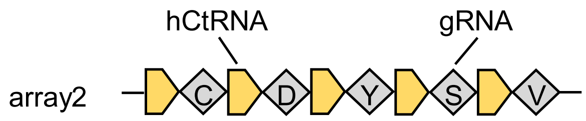

Supplementary Figure 8. 5-loci hCtRNA-M array for comparison with Cas12a MBE strategy. hU6**,** human U6 promoter**.** C, CDKN2A; D, DNMT1; Y, DYRK1A; S, SITE3; V, VEGFA. 7-bp poly-T termination signal is not shown.

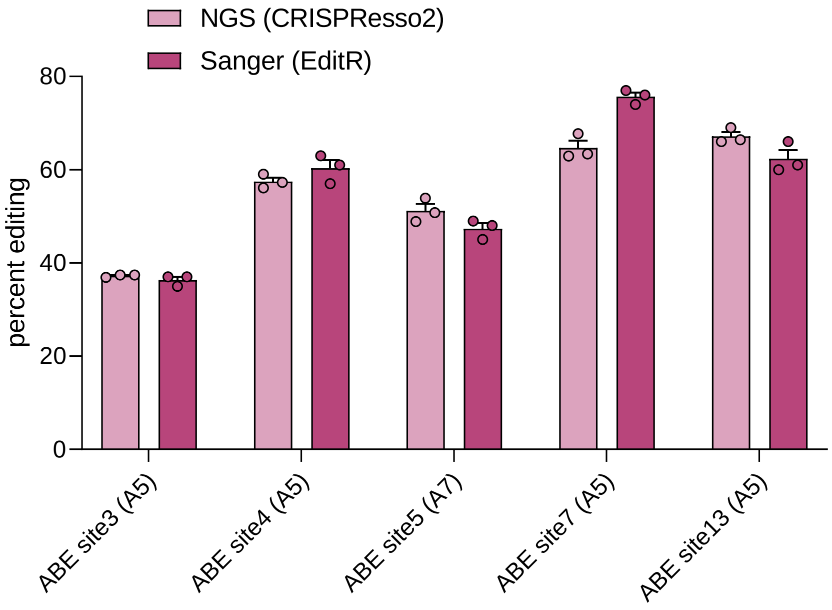

Supplementary Figure 9. Comparison of EditR to CRISPResso2 in evaluating the 5-loci MBE efficiencies. ABE 7.10 (F148A) and 5-loci hCtRNA-M array were transfected in HEK293T cells. Genomic DNA was extracted 3 days post-transfection, the same sample was used to generate amplicons for Sanger sequencing (EditR) and NGS (CRISPResso2), respectively. Error bars represent mean ± s.e.m. from n = 3 replicates.

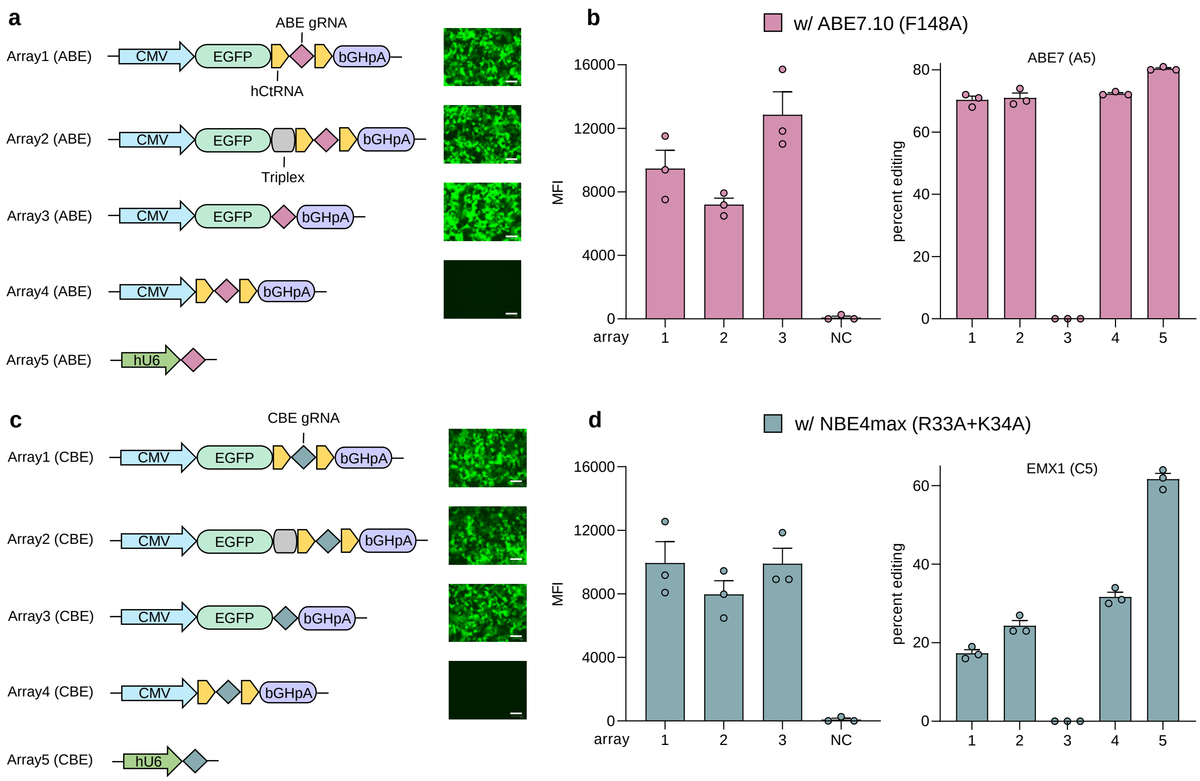

Supplementary Figure 10. Single transcript architecture containing protein-coding sequence and hCtRNA-gRNA array driven by an upstream RNA Pol II promoter. a,**c**, Schematic of single transcripts designed for ABE (**a**) and CBE (**c**). Representative fluorescent images showing EGFP expressed by corresponding transcripts in HEK293T cells. CMV, human cytomegalovirus promoter (RNA Pol II). Triplex, a tertiary RNA structural motif. bGHpA, bovine growth hormone poly-A termination signal. Scale bar, 100 μm. **b**,**d**, Evaluation of fluorescence intensity and BE efficiency of designed single transcripts (co-transfected with the indicated base editor) in HEK293T cells. MFI, median fluorescent intensity. NC, negative control. NC data of **b** is the same as in **d**. Error bars represent mean ± s.e.m. from n = 3 replicates.

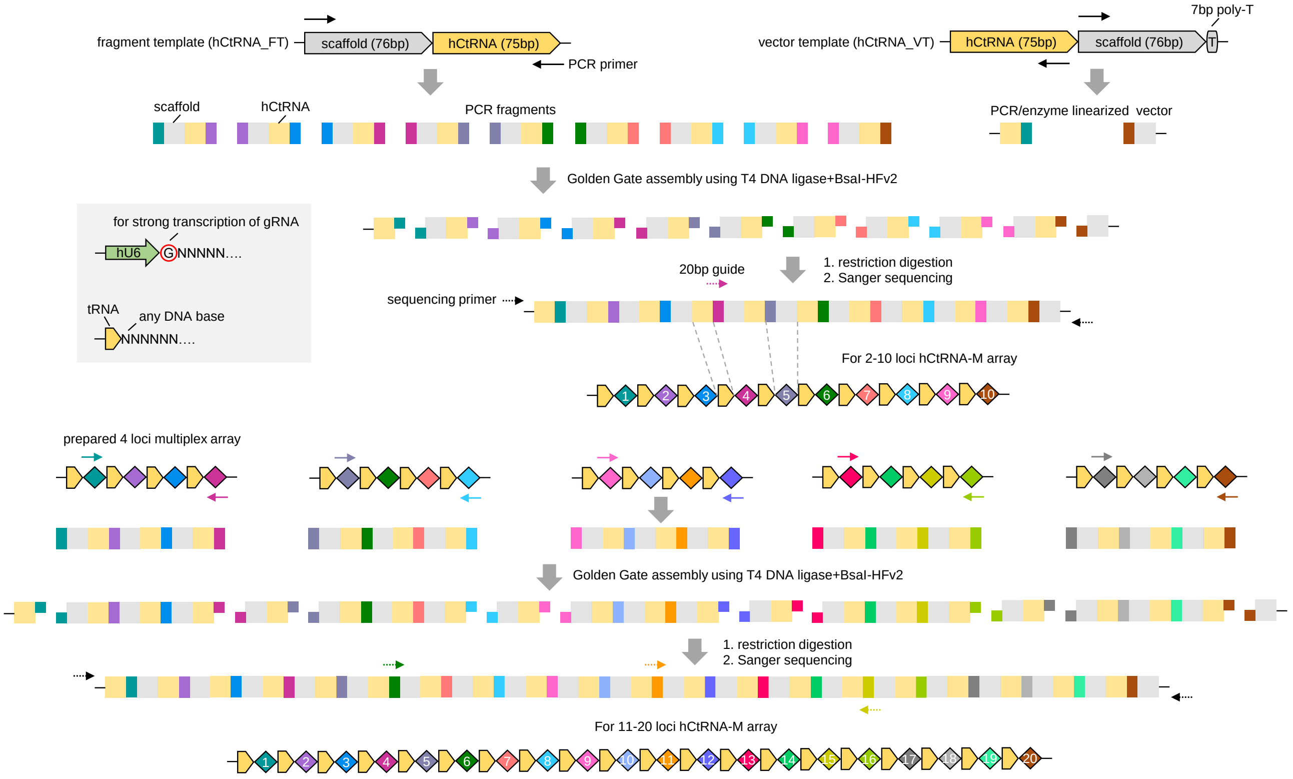

Supplementary Figure 11. Construction and verification of customized hCtRNA-M array. Compared to the traditional U6 promoter that prefers a 5' guanosine for robust transcription of gRNAs, which can cause a mismatch between the target sequence and gRNA and will affect the performance of some high-fidelity Cas variants, the hCtRNA-M array lacks the sequence restriction for gRNA design, enabling the gRNA to perfectly match the target sequence. To construct hCtRNA-M arrays for multiplex gene editing in mammalian cells, hCtRNA_FT (Addgene) is used as a template to amplify fragments flanked by partial guide sequence and recognition sequence of BsaI-HFv2 (New England Biolabs) for downstream Golden Gate assembly. hCtRNA_VT (Addgene) is used to provide a linearized vector to assemble the hCtRNA-M array completely. The recommended annealing temperatures for sufficiently amplifying the fragment and vector are 51-55°C and 60°C, respectively. The hCtRNA-M array was verified by restriction digestion and Sanger sequencing. Protospacer sequences were used as Sanger sequencing primers. Example cloning design of MBE array; Example cloning design of MPE array.

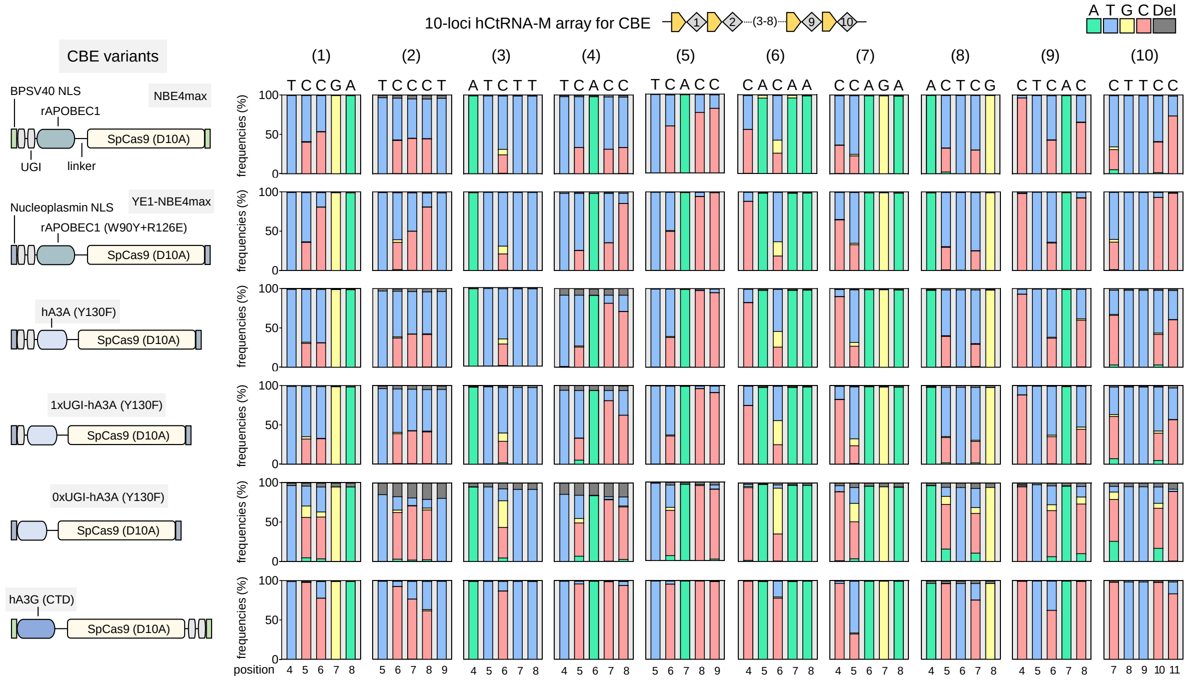

Supplementary Figure 12. On-target MBE activities of 6 CBE variants with 10-loci hCtRNA-M array. Bar plots showing 10-loci MBE frequencies of 6 CBE variants tested in HEK293T cells. The short name of each CBE variant is highlighted with a gray background. hA3G (CTD)[1], C-terminal catalytic domain of human A3G deaminase. Del, deletions at each nucleotide. Bottom numbers indicate the position of the base in the protospacer, counting 1 as the most PAM-distal base. Mean values of n = 3 replicates are shown in each bar. Detailed protospacer and amplicon information are available in **Supplementary Table 5**. Heatmap characterizations of hA3A (Y130F), 1xUGI-hA3A (Y130F), 0xUGI-hA3A (Y130F) and hA3G (CTD) edits are shown in **Supplementary Fig. 15**. Heatmap characterization of MBE using 1xUGI-hA3A (Y130F) and 10-loci hCtRNA-M designed for ABE is shown in **Supplementary Fig. 16a**.

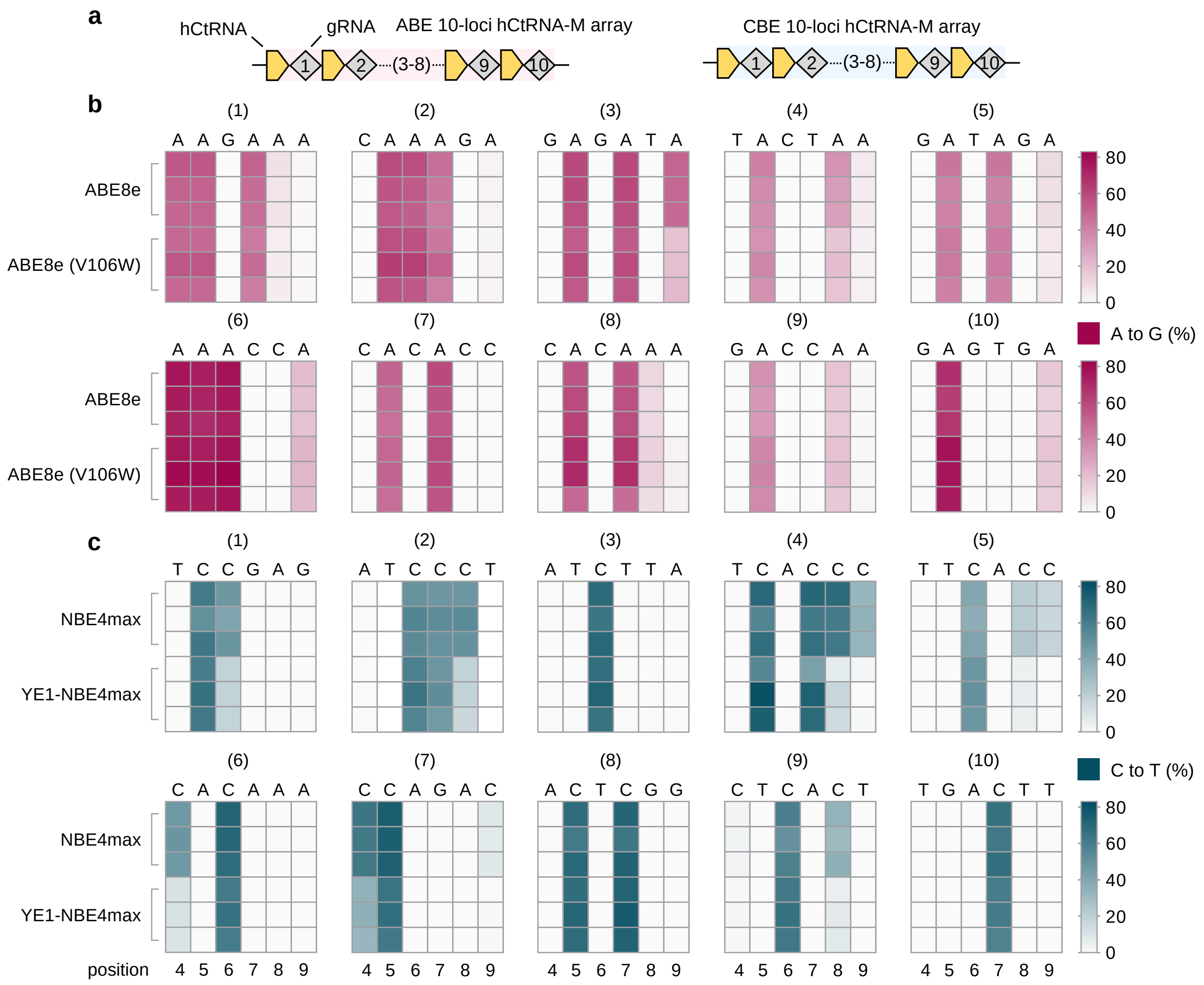

Supplementary Figure 13. Additional characterization of ABE8e and NBE4max variants for MBE with 10-loci hCtRNA-M array in HEK293T cells. a, Schematic of two 10-loci hCtRNA-M arrays used for multiplex ABE and CBE, respectively. **b**,**c**, Heat maps showing the on-target DNA A-to-G (plum) and C-to-T (dark teal) percent editing, with 3 replicates for each base editor variant. ABE8e (V106W), ABE8e with TadA-8e V106W mutation. The bottom numbers represent the DNA base positions within the protospacer, counting 1 as the most PAM-distal position. The editing windows shown (positions 4 to 9) represent the most highly edited region. Protospacer and amplicon information are available in **Supplementary Table 5**. Heatmap characterization of MBE using ABE8e (V106W) and 10-loci hCtRNA-M designed for CBE is shown in **Supplementary Fig. 16b**.

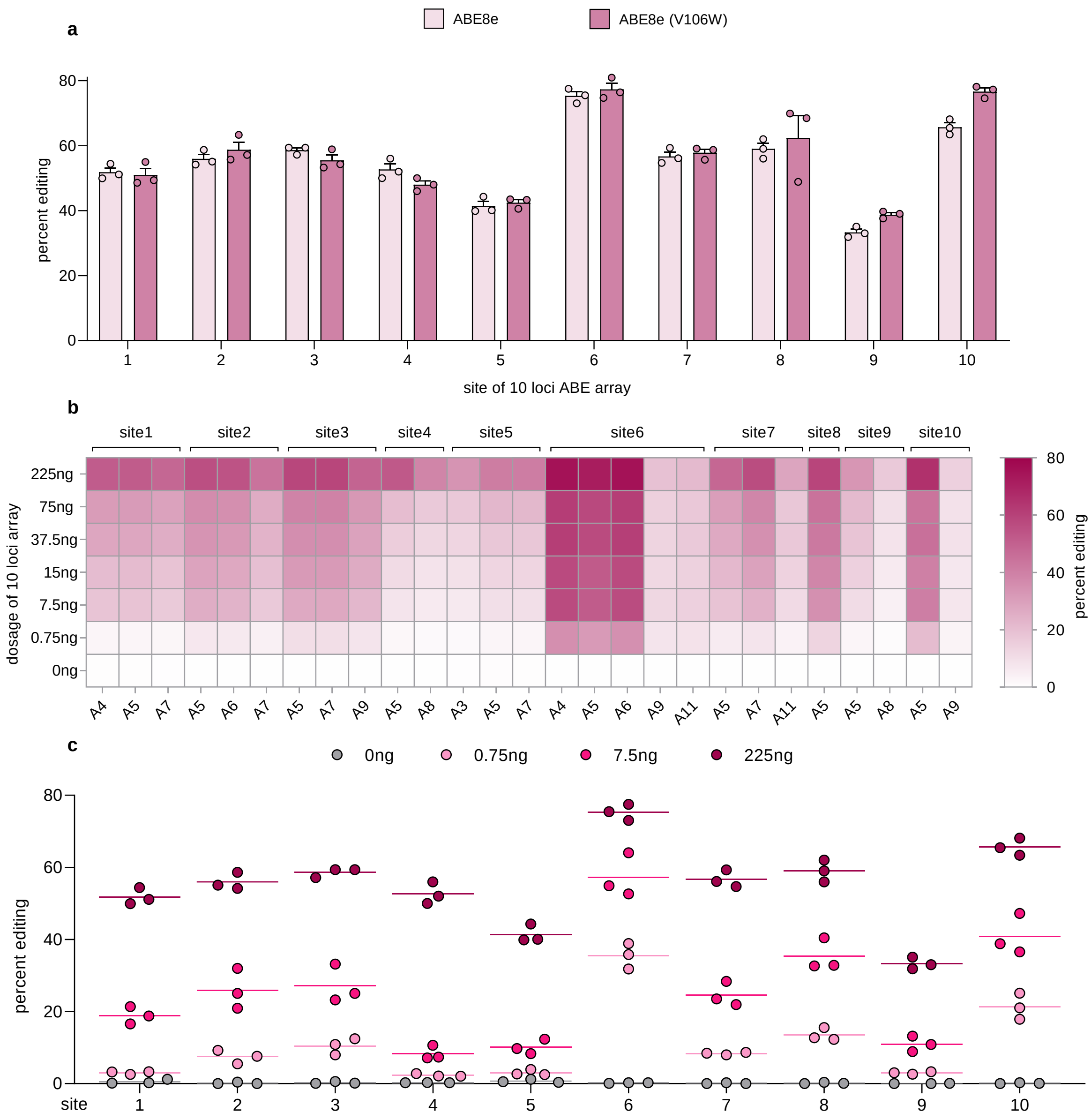

Supplementary Figure 14. 10-loci MBE in HEK293T cells using hCtRNA-M array and ABE8e variants. a, Comparing 10-loci MBE performance of ABE8e and ABE8e (V106W). Error bars represent mean ± s.e.m. from n = 3 replicates. **b**, Heat maps showing MBE using ABE8e and 10-loci hCtRNA-M array of different doses. Base editing efficiencies were analyzed by NGS (CRISPResso2). **c**, MBE using ABE8e and 10-loci hCtRNA-M array of different doses. The amount of ABE8e is constant 225 ng in each dose group. No filler plasmid was used. Line values of scatter dot plot represent the mean of three biological replicates.

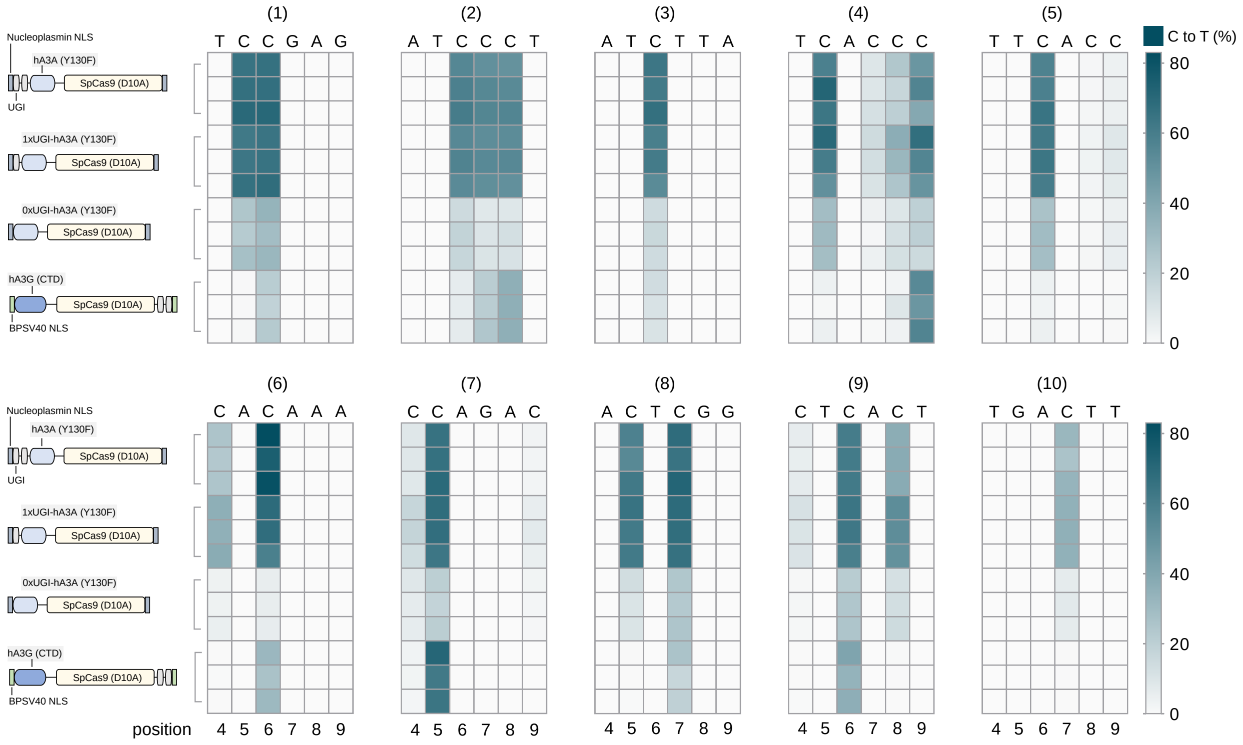

Supplementary Figure 15. Additional characterization of 10-loci MBE using hA3A and hA3G CBE variants and hCtRNA-M array in HEK293T cells. Heat maps showing the percent on-target C-to-T editing with n = 3 replicates at each site.

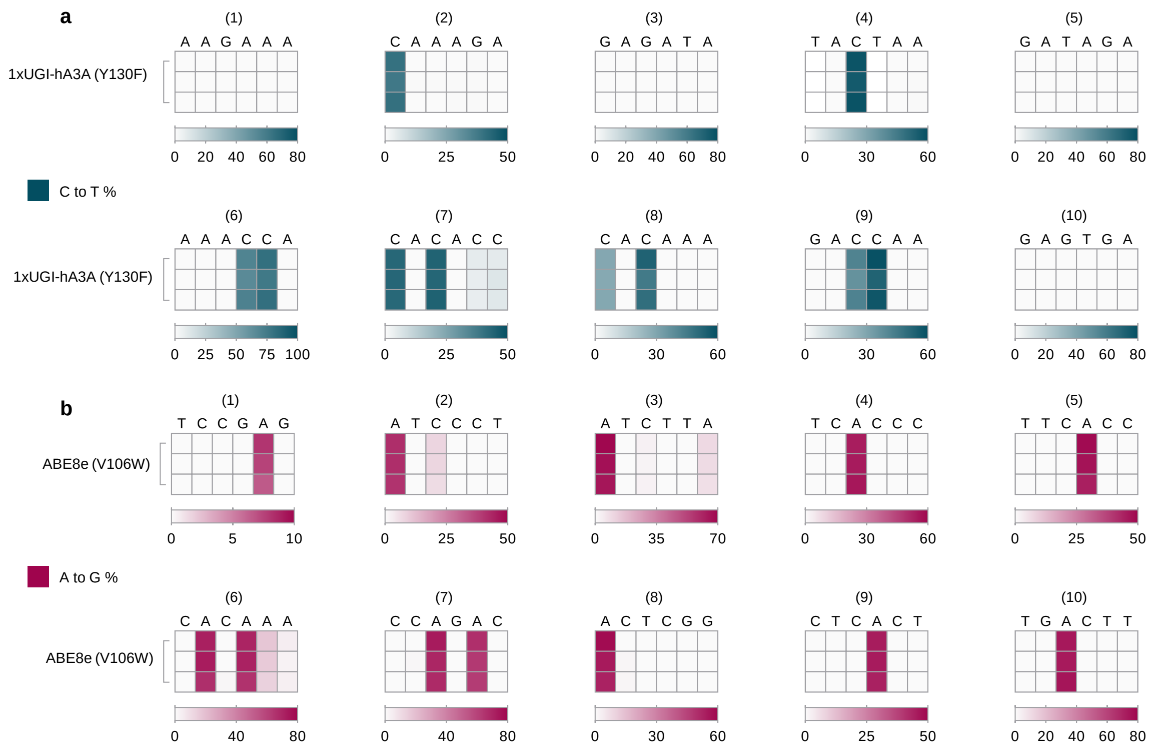

Supplementary Figure 16. Additional characterization of 10-loci MBE using ABE8e (V106W), 1xUGI-hA3A (Y130F) and hCtRNA-M arrays in HEK293T cells. Heat maps showing multiplex C-to-T editing (**a**) and A-to-G editing (**b**) with n = 3 replicates at each site. ABE8e (V106W) and 1xUGI-hA3A (Y130F) were co-transfected with 10-loci hCtRNA-M arrays originally designed for CBE and ABE, respectively.

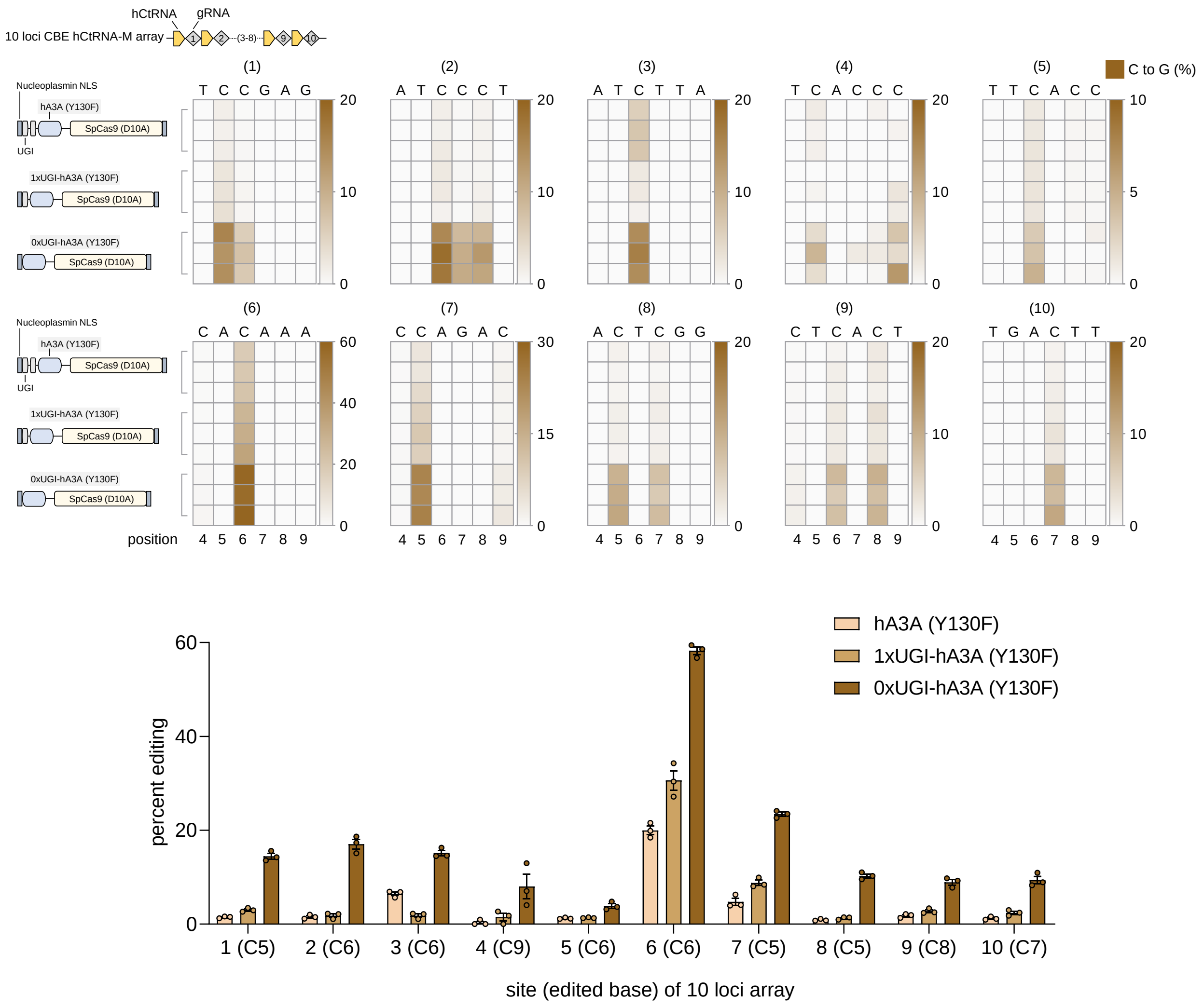

Supplementary Figure 17. Multiplex C-to-G base editing in HEK293T cells. Heat maps with three replicates showing 10-loci multiplex C-to-G editing by hA3A (Y130F), 1xUGI-hA3A (Y130F) and 0xUGI-hA3A (Y130F). Error bars represent mean ± s.e.m. from n = 3 replicates.

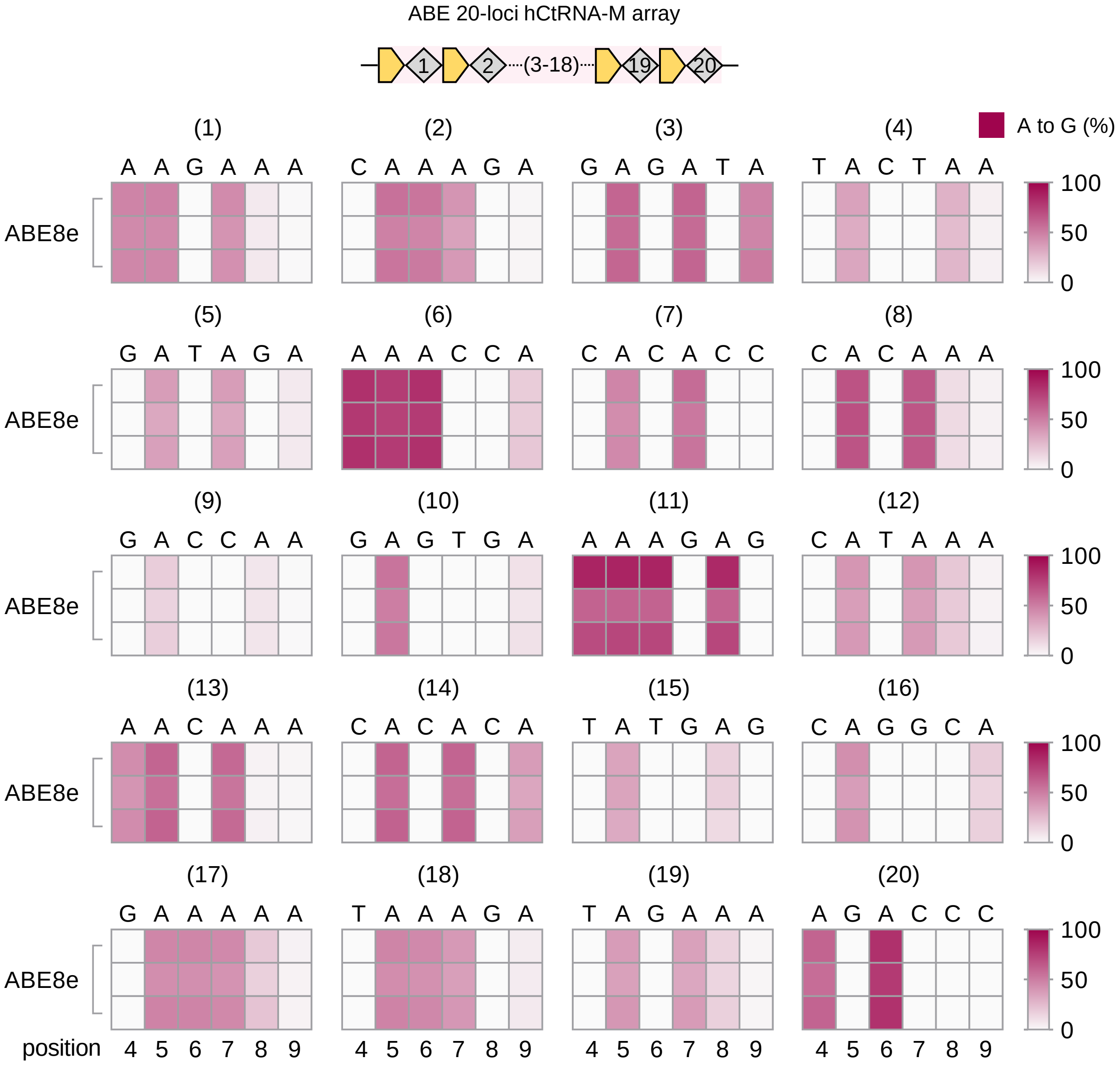

Supplementary Figure 18. 20-loci MBE with ABE8e and hCtRNA-M array in HEK293T cells. Heat maps showing the percent on-target A-to-G (plum) editing with n = 3 replicates at each site. The protospacer and amplicon information is available in **Supplementary Table 5**.

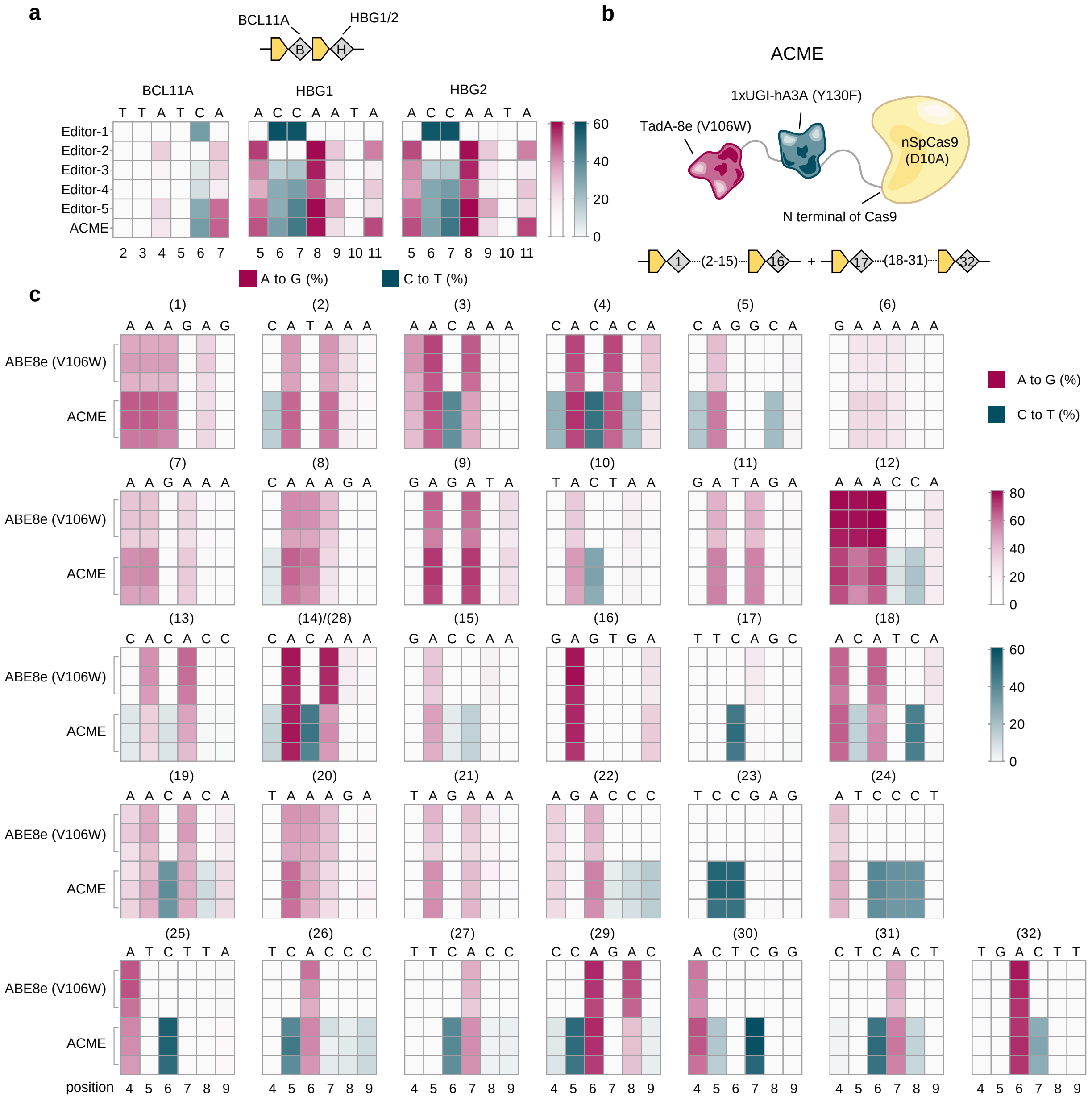

Supplementary Figure 19. 31-loci MBE with ACME and pooled hCtRNA-M arrays in HEK293T cells. a, Heat maps comparing the on-target MBE of 6 base editor variants at BCL11A and HBG1/2 loci. Editor-1, YE1-NBE4max; Editor-2, ABE8e (V106W); Editor-3, dual-deaminase base editor with 2xUGI-rAPOBEC1 (YE1)-TadA-8e (V106W) deamination module at N-terminal of Cas9; Editor-4, dual-deaminase base editor with TadA-8e (V106W)-2xUGI-rAPOBEC1 (YE1) deamination module at N-terminal of Cas9; Editor-5, dual-deaminase base editor with 1xUGI-hA3A (Y130F)-TadA-8e (V106W) deamination module at N-terminal of Cas9; ACME, dual-deaminase base editor with TadA-

8e (V106W)-1xUGI-hA3A (Y130F) deamination module at N-terminal of Cas9. A to G or C to T conversion within the indicated editing windows has been shown to induce the upregulation of fetal hemoglobin (HbF) and could be a promising therapeutic strategy in treating SCD and β-thalassemia. **b**, Schematic of ACME and two 16-loci hCtRNA-M arrays. **c**, Heat maps showing percent editing of A-to-G (plum) and C-to-T (dark teal) by ABE8e (V106W) and ACME, with 3 replicates for each editor. Number 14 and 28 gRNA of hCtRNA-M arrays are identical. The bottom numbers represent the DNA base positions within the protospacer, counting 1 as the most PAM-distal position. The editing windows shown (positions 4 to 9) represent the most highly edited region. The protospacer and amplicon information are available in **Supplementary Table 5**. ACME plasmid map is available in **Supplementary Table 1**.

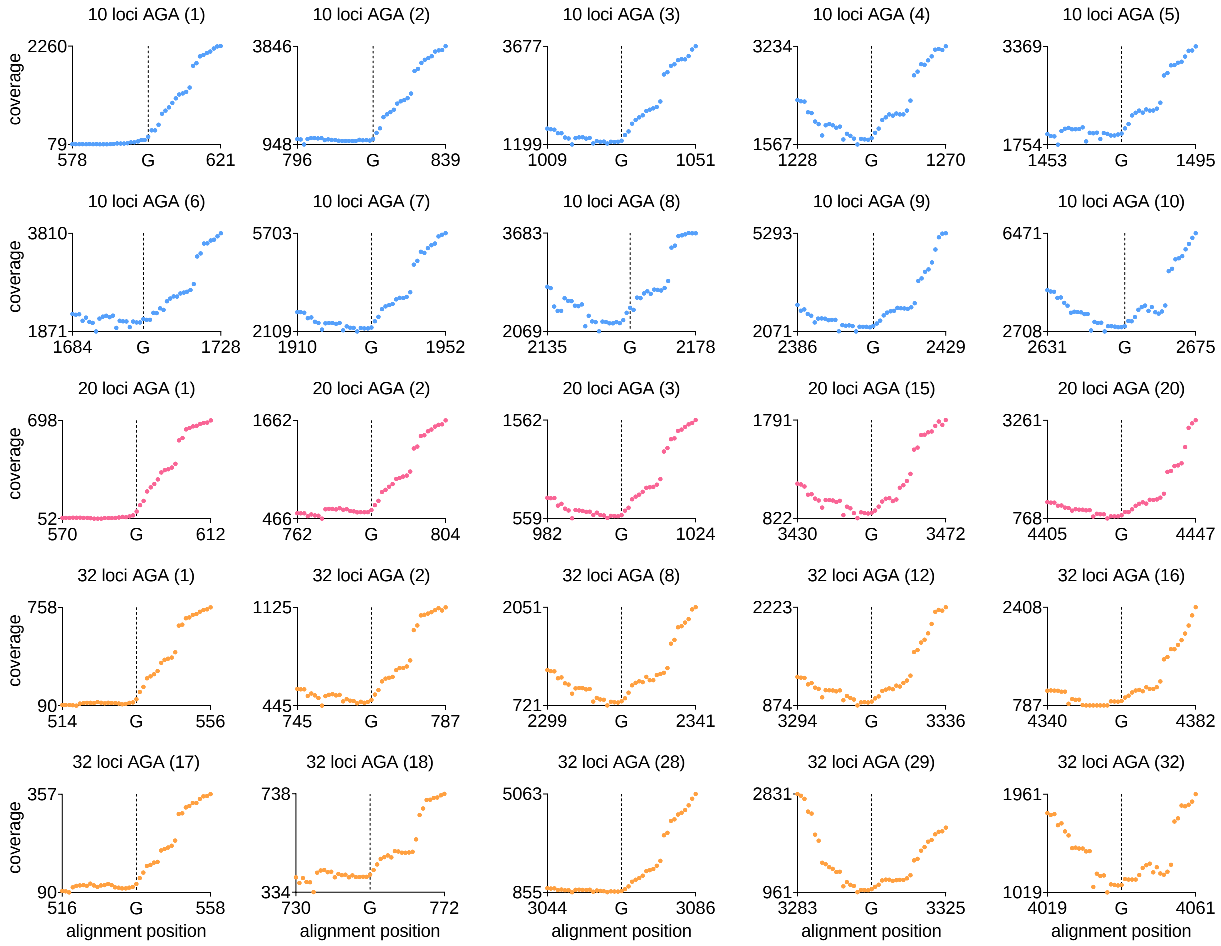

Supplementary Figure 20. RNA-seq information at 5' leader region of hCtRNA-M arrays. Selected RNA-seq alignments surrounding the AGA 5' leader sequence of hCtRNA. The explanation of each plot, for examples, 10 loci AGA (1), depicts the hCtRNA at the left side of the number 1 gRNA in the 10-loci hCtRNA-M array shown in **Fig. 3a**; 20 loci AGA (15) profiles the hCtRNA at the left side of the number 15 gRNA in the 20-loci hCtRNA-M array shown in **Fig. 3b**; 32 loci AGA (32) characterizes the hCtRNA at the left side of the number 32 gRNA in the 16-loci hCtRNA-M array shown in **Fig. 3c**. Note, two of the gRNAs are identical, so our total editing is 31 different loci. The "G" of the "AGA" 5' leader sequence is centered on the X-axis. The lower values of the alignment position represent the more upstream position of the specified hCtRNA. The gRNA with more upstream hCtRNAs shows higher coverage than the gRNA with less upstream hCtRNAs.

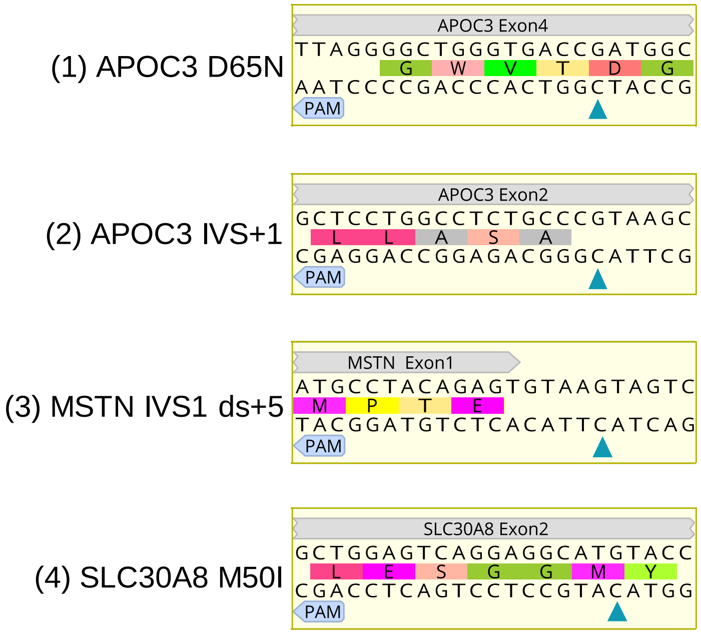

Supplementary Figure 21. Schematic of target sites used for multiplex installation of protective genetic variants against polygenic diseases. Dark teal arrow indicating the edited DNA base.

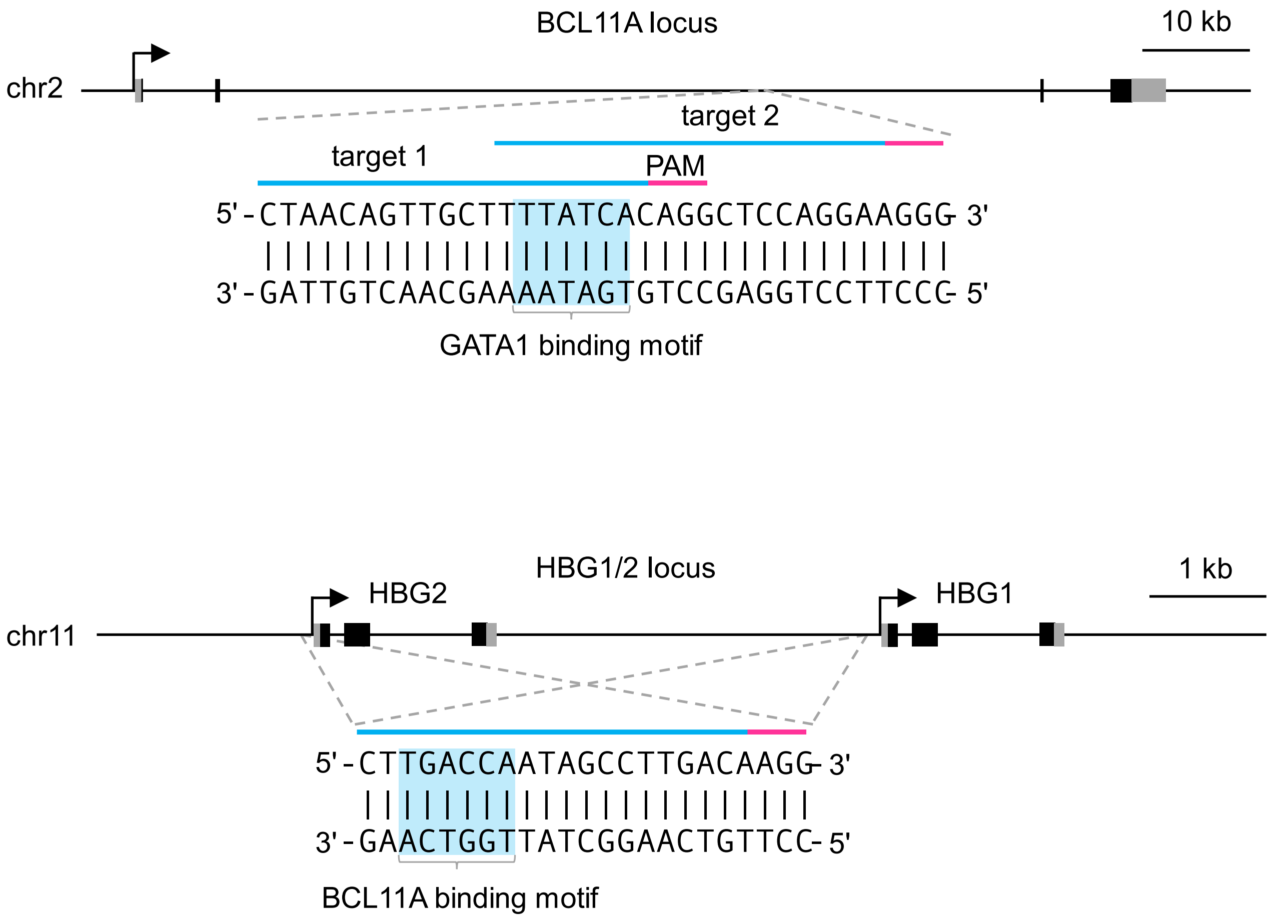

Supplementary Figure 22. Schematic of human BCL11A and HBG1/2 loci showing the location of three protospacers indicated by blue lines with PAM in magenta. Target 1 is used for Cas9 nuclease editing. Target 2 is used for base editing.

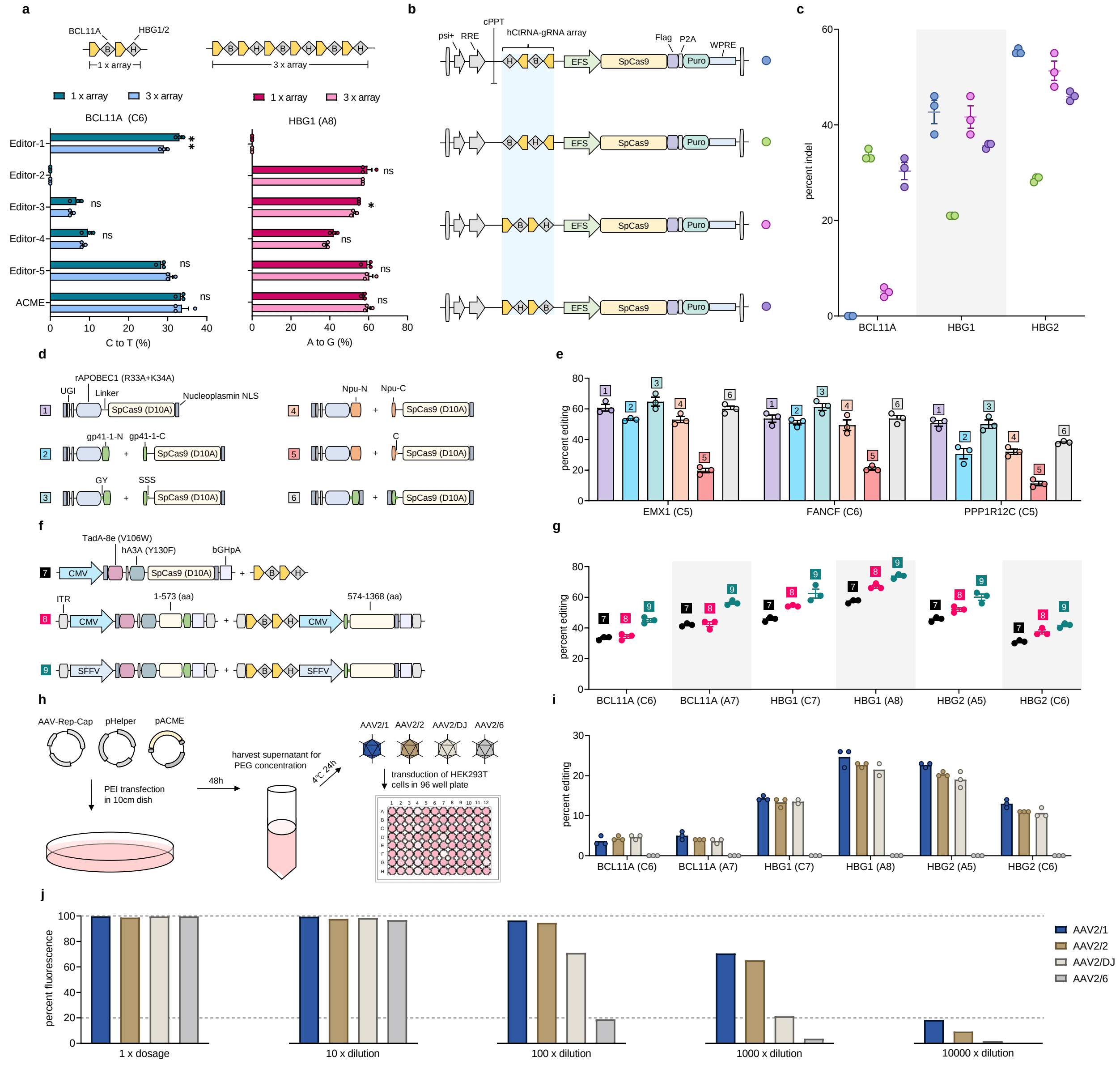

Supplementary Figure 23. Adapting hCtRNA-M array to AAV and lentiviral delivery. a, Two gRNAs targeting BCL11A and HBG1/2 loci were assembled as 1 x or 3 x array. Tested by six base editor variants depicted in **Supplementary Fig. 19a and b**. Unpaired two-tailed t-test was performed (ns, not significant; *P < 0.05; **P < 0.01; ***P < 0.001; ****P < 0.0001). **b**,**c**, 1 x arrays of all possible directions and orders were cloned into lentiCRISPRv2 vector by replacing the original hU6 cassette and tested for multiplex genome editing in HEK293T cells. From top to bottom, the second construct (green dot) and the fourth (purple dot) were packaged as lentivirus for comparison in **Fig. 5e**. WPRE, Woodchuck hepatitis virus post-transcriptional regulatory element; Puro, puromycin selection

marker; P2A, porcine teschovirus-1 2A self-cleaving peptide; Flag, Flag octapeptide tag; cPPT, central polypurine tract; RRE, Rev response element; psi+, psi packaging signal. **d**, **e**, Optimization of the split BE. Npu-N/C N- or C-terminal Npu trans-splicing intein; C, extein amino acid residue Cys. The trans-splicing intein, extein, and NLS architecture of the number 3 split base editor were used in the following designs. gRNAs used in **e** were driven by hU6 protomer and tested by single-delivery for each site. **f**,**g**, ACME split into two AAV vectors without affecting the dual BE efficiencies. Split ACME was driven by SFFV promoter achieving higher editing efficiencies. **h**, Schematic of the developed process for AAV production and concentration. Four pseudotypes of ACME dual AAV were packaged using the number 9 construct in **f**. **i**, HEK293T cells were transduced by dual AAVs of four pseudotypes. **j**, Comparing the transduction efficiencies of four pseudotype AAV packaged using a vector expressing EGFP. 1 x dosage, 10 µl of concentrated AAV. Approximately 2 x 10^4 HEK293T cells were transduced in each well of the 96-well plate for 2 days before flow cytometry analysis. Error bars represent mean ± s.e.m. from n = 3 replicates. Gating strategy is shown in **Supplementary Figure 30.**

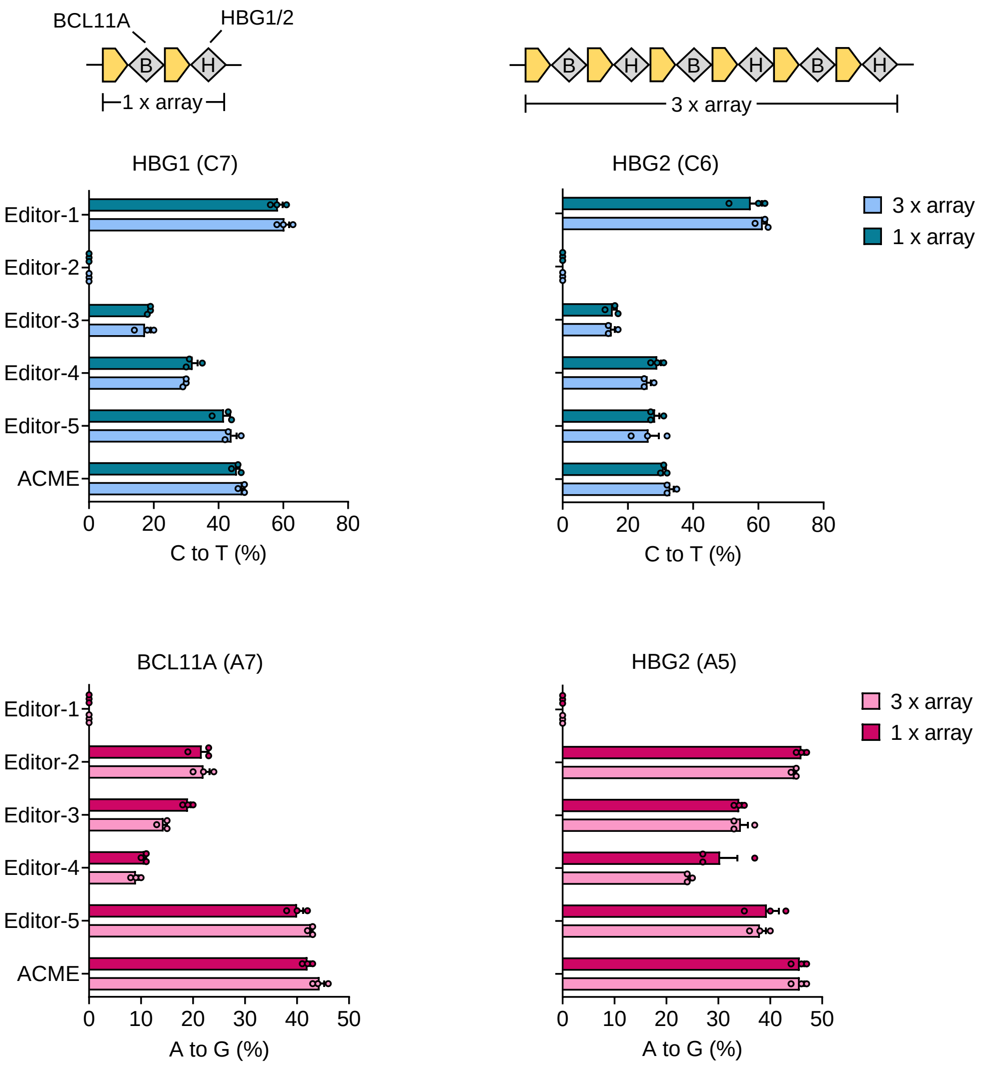

Supplementary Figure 24. Additional MBE comparison of 1 x array and 3 x array as depicted in Supplementary Fig. 23a. Error bars represent mean ± s.e.m. from n = 3 replicates.

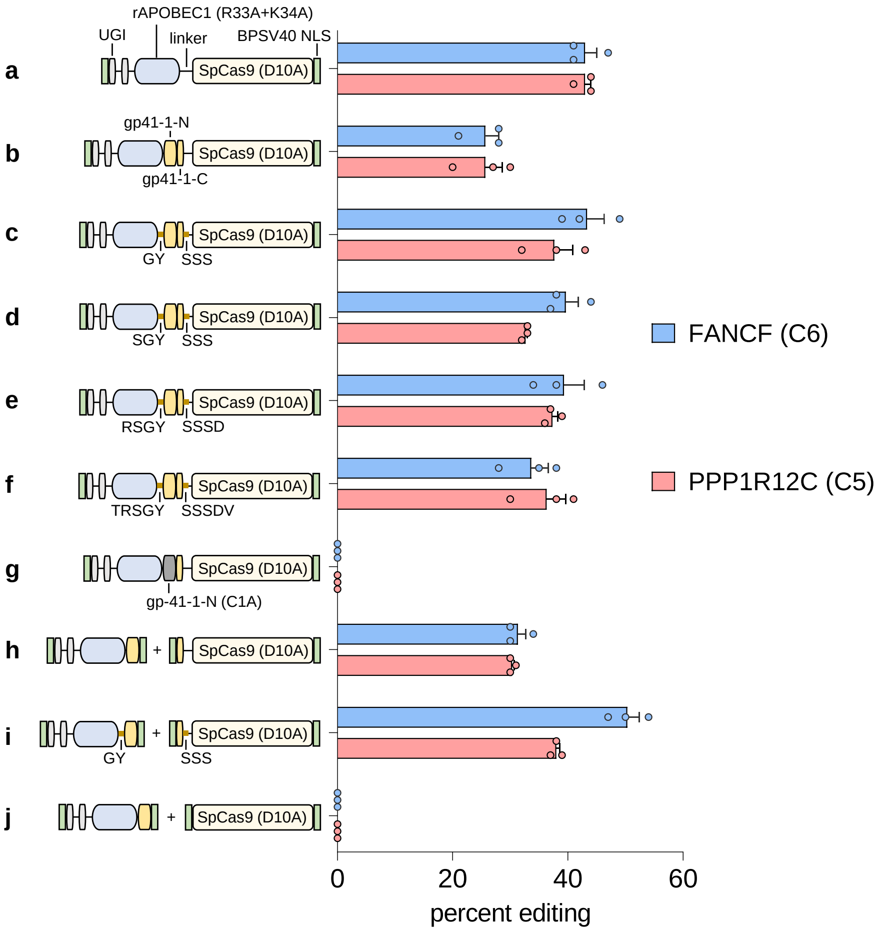

Supplementary Figure 25. Split base editor using gp41-1 trans-splicing intein and extein flanking the split site. a, Full-length NBE4max (R33A+K34A). **b**, Cis-splicing NBE4max (R33A+K34A) with full-length gp41-1 trans-splicing intein inserted at the linker region. gp41-1-N/C, N- or C-terminal gp41-1 trans-splicing intein. **c**, Cis-splicing NBE4max (R33A+K34A) with full-length gp41-1, GY, and SSS extein at the linker region. Abbreviations for the amino acid residues: A, Ala; C, Cys; D, Asp; E, Glu; F, Phe; G, Gly; H, His; I, Ile; K, Lys; L, Leu; M, Met; N, Asn; P, Pro; Q, Gln; R, Arg; S, Ser; T, Thr; V, Val; W, Trp; and Y, Tyr. **d**, Cis-splicing NBE4max (R33A+K34A) with full-length gp41-1, SGY, and SSS extein at the linker region. **e**, Cis-splicing NBE4max (R33A+K34A) with full-length gp41-1, RSGY, and SSSD extein at the linker region. **f**, Cis-splicing NBE4max (R33A+K34A) with full-length gp41-1, TRSGY, and SSSDV extein at the linker region. **g**, Negative control of Cis-splicing NBE4max (R33A+K34A) with full-length gp41-1 (C1A). C1A mutation inactivates the splicing reaction of gp41-1 by disabling the cleavage at gp41-1-N, while the gp41-1-

C can still be released, separating the rAPOBEC1 (R33A+K34A) from SpCas9 (D10A). **h**, Trans-splicing NBE4max (R33A+K34A) with full-length gp41-1 trans-splicing intein inserted at the linker region and 4 NLS. **i**, Trans-splicing NBE4max (R33A+K34A) with full-length gp41-1, GY, and SSS extein at the linker region. **j**, Negative control of trans-splicing NBE4max (R33A+K34A) with SpCas9 (D10A) lacking the gp41-1-C part. Base editing efficiencies were analyzed by Sanger sequencing. Error bars represent mean ± s.e.m. from n = 3 replicates.

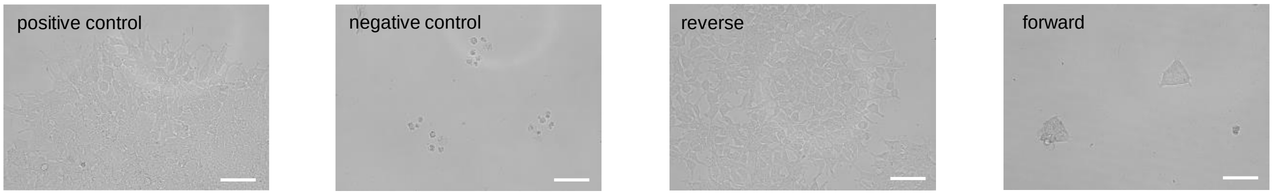

Supplementary Figure 26. Reverse hCtRNA-M array on lentiviral vector enables effective transduction in human cells. Representative images of HEK293T cells 7 days post-transduction by lentiviruses containing the reverse or forward hCtRNA-M array, puromycin selection was applied 24 h after transduction. Positive control, w/o transduction and puro-selection. Negative control, w/o transduction but w/ puro-selection. Scale bar, 100 µm. Three times of repeats was performed independently with similar results for each condition shown above.

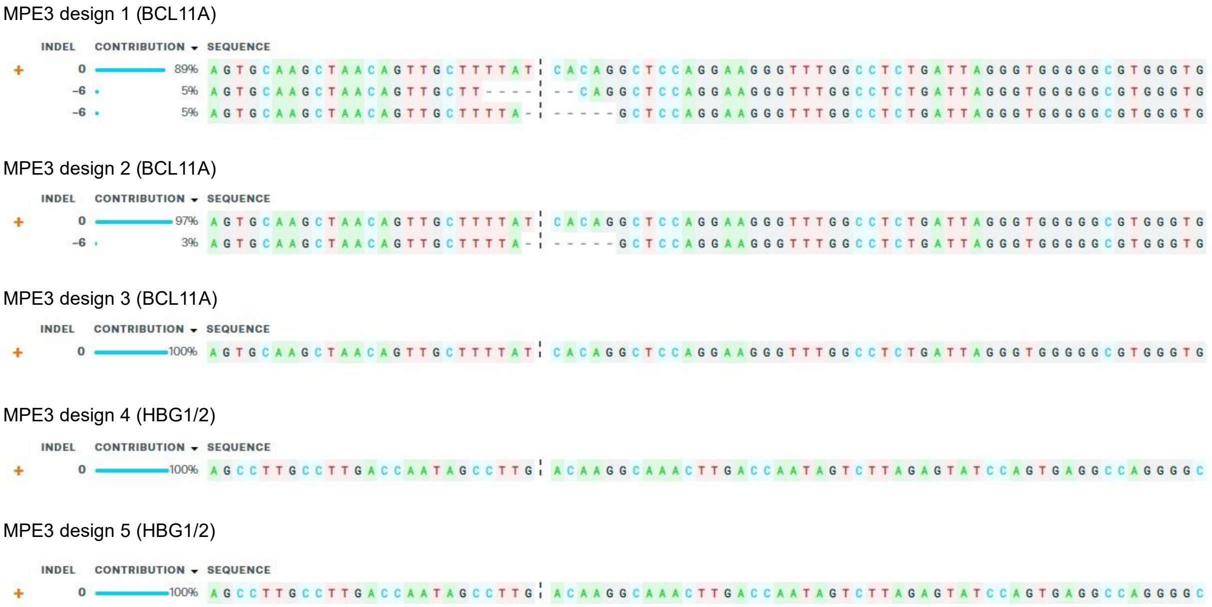

Supplementary Figure 27. One out of five designed MPE3 arrays enables substantial deletions. Sanger sequencing results were analyzed using ICE webtool (https://ice.synthego.com/)[2]. MPE3 design 1 (BCL11A) was used to construct the gene cassette for the lentiviral delivery experiments.

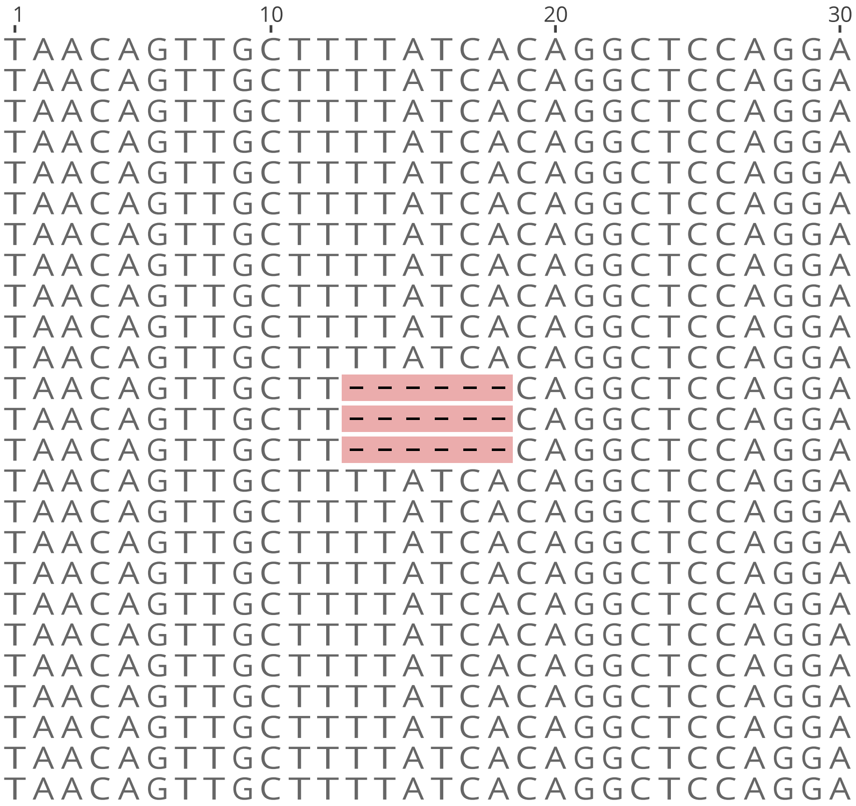

Supplementary Figure 28. Representative desired MPE3 reads from lentiviral transduced K562 cells. The deletion of TTATCA shown in red represents desired MPE3 editing of the GATA1 motif at the BCL11A site.

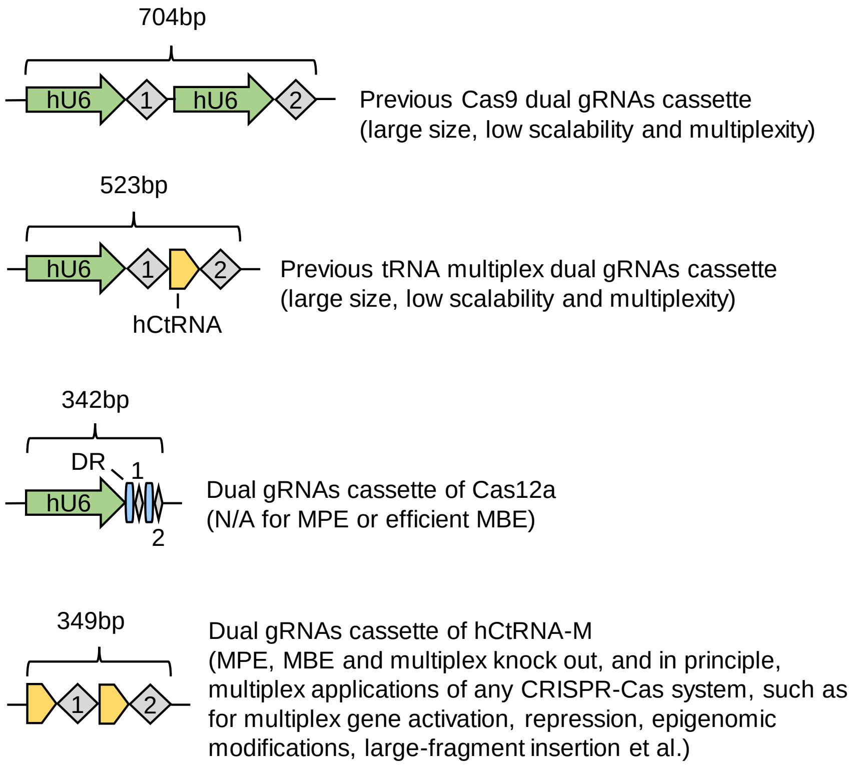

Supplementary Figure 29. Comparison of hCtRNA-M with current DNA cassettes for dual editing in multiplex CRISPR screening applications[3, 4]. DR, direct repeat.

Supplementary Figure 30. Example flow cytometry gating strategy.

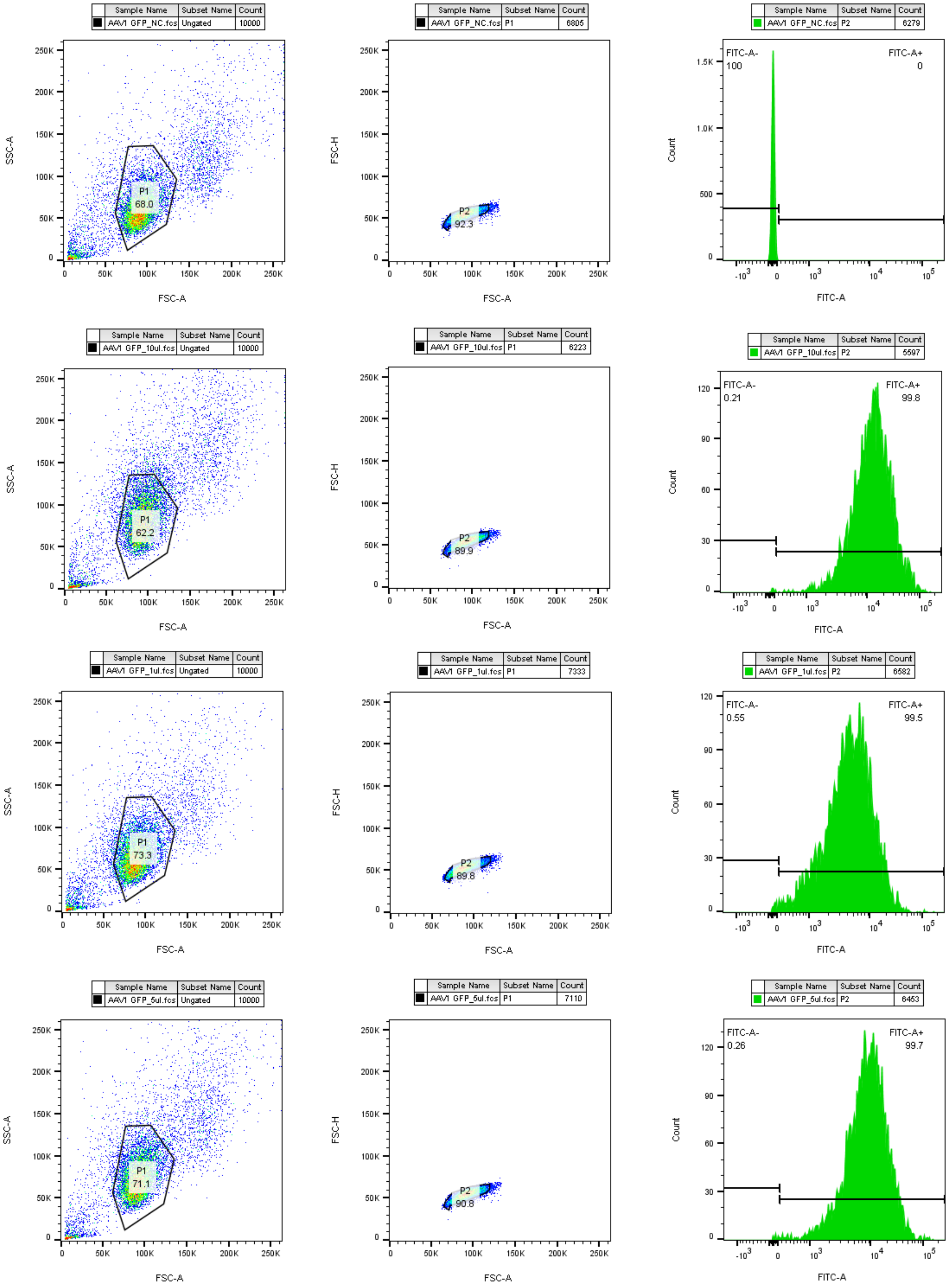

Supplementary Table 1. Representative plasmids used in this study.

Plasmid name	Brief description	Benchling links
ABE-dLbCas12a	Nucleoplasmin NLS-TadA-8e-dLbCas12a-Nucleoplasmin NLS	link
CBE-dLbCas12a	Nucleoplasmin NLS-2xUGI-rAPOBEC1-dLbCas12a-Nucleoplasmin NLS	link
array1	5-loci hU6-driven multiplex array of dLbCas12a base editors (comparing with nSpCas9 base editors)	link
hCtRNA_FT	fragment template (FT) of hCtRNA multiplex array	link
hCtRNA_VT	vector template (VT) of hCtRNA multiplex array	link
array2	5-loci hCtRNA-M array for nSpCas9 base editors (comparing with dLbCas12a base editors)	link
Multiplex_lentiCRISPR v2	example of hCtRNA multiplex array on lentiCRISPR v2 backbone targeting BCL11A and HBG1/2 loci	link
MPE_lentiCRISPR v2	targeting BCL11A enhancer to delete GATA1 binding motif	link
pCMV-ACME	full-length ACME driven by CMV promoter	link
pAAV-ACME-IntN	partial ACME dual-deaminase base editor split by gp41-1 intein N-termini with GY extein	link
pAAV-ACME-IntC	hCtRNA multiplex array and partial ACME dual-deaminase base editor split by gp41-1 intein C-termini with SSS extein	link
10-loci hCtRNA-M_CBE	10-loci hCtRNA-M array designed for CBE	link
10-loci hCtRNA-M_ABE	10-loci hCtRNA-M array designed for ABE	link
3-loci hCtRNA-M _PE3	3-loci hCtRNA-M array encoding 3 pairs of nicking gRNA and pegRNA with interval sequence (I) for PE3	link
20-loci hCtRNA-M_ABE	20-loci hCtRNA-M array designed for ABE8e	link

16-loci hCtRNA-M_01_ACME	16-loci hCtRNA-M array designed for 31-loci MBE using ACME	link
16-loci hCtRNA-M_02_ACME	16-loci hCtRNA-M array designed for 31-loci MBE using ACME	link
NBE4max	BPSV40-2XUGI-rAPOBEC1-nCas9-BPSV40	link
NBE4max-SpRY	Nucleoplasmin NLS-2xUGI-rAPOBEC1-nSpRY-Nucleoplasmin NLS	link
hA3A (Y130F)	Nucleoplasmin NLS-2xUGI-hA3A (Y130F)-nCas9-Nucleoplasmin NLS	link
YE1-NBE4max	Nucleoplasmin NLS-2xUGI-rAPOBEC1 YE1-nCas9-Nucleoplasmin NLS	link
0xUGI-hA3A (Y130F)	Nucleoplasmin NLS-hA3A (Y130F)-nCas9-Nucleoplasmin NLS	link
1xUGI-hA3A (Y130F)	Nucleoplasmin NLS-1xUGI-hA3A (Y130F)-nCas9-Nucleoplasmin NLS	link
Editor-3	2xUGI-rAPOBEC1 (YE1)-TadA-8e (V106W)-nCas9 dual-deaminase base editor	link
Editor-4	TadA-8e (V106W)-2xUGI-rAPOBEC1 (YE1)-nCas9 dual-deaminase base editor	link
Editor-5	1xUGI-hA3A (Y130F)-TadA-8e (V106W)-nCas9 dual-deaminase base editor	link

Supplementary Table 2. List of mature tRNA variants tested in this work.

Name	Sequence	tRNA count	tRNA database link
hCys GCA	GGGGGTATAGCTCAGTGGTAGAGCATTTGACTGCAGATCAAGAGGTCCCCGGTTCAAATCCGGGTGCCCCCT	29	link
hAla AGC	GGGGGTATAGCTCAGTGGTAGAGCGCGTGCTTAGCATGCACGAGGTCCTGGGTTCGATCCCCAGTACCTCCA	26	link
hAsn GTT	GTCTCTGTGGCGCAATCGGTTAGCGCGTTCGGCTGTTAACCGAAAGGTTGGTGGTTCGAGCCCACCCAGGGACG	25	link
hLys CTT	GCCCGGCTAGCTCAGTCGGTAGAGCATGAGACTCTTAATCTCAGGGTCGTGGGTTCGAGCCCCACGTTGGGCG	15	link
hIle AAT	GGCCGGTTAGCTCAGTTGGTTAGAGCGTGGTGCTAATAACGCCAAGGTCGCGGGTTCGATCCCCGTACTGGCCA	15	link
hGly GCC	GCATTGGTGGTTCAGTGGTAGAATTCTCGCCTGCCACGCGGGAGGCCCGGGTTCGATTCCCGGCCAATGCA	14	link
hGln CTG	GGTTCCATGGTGTAATGGTTAGCACTCTGGACTCTGAATCCAGCGATCCGAGTTCAAATCTCGGTGGAACCT	13	link
AtGly GCC	GCACCAGTGGTCTAGTGGTAGAATAGTACCCTGCCACGGTACAGACCCGGGTTCGATTCCCGGCTGGTGCA	21	link

Supplementary Table 3. List of tRNA variants with 5' leader sequence.

Name	Sequence (5' leaders with optimized length for multiplex editing are colored in red)	tRNA database link
hCtRNA	AGAGGGGGTATAGCTCAGTGGTAGAGCATTTGACTGCAGATCAAGAGGTCCCCGGTTCAAATCCGGGTGCCCCCT	link
AtGly GCC	AACAAAGCACCAGTGGTCTAGTGGTAGAATAGTACCCTGCCACGGTACAGACCCGGGTTCGATTCCCGGCTGGTGCA	link
hGln CTG	GGCGGTTCCATGGTGTAATGGTTAGCACTCTGGACTCTGAATCCAGCGATCCGAGTTCAAATCTCGGTGGAACCT	link
hGly GCC	GCTGGAGCATTGGTGGTTCAGTGGTAGAATTCTCGCCTGCCACGCGGGAGGCCCGGGTTCGATTCCCGGCCAATGCA	link
hIle AAT	TCATCCGGCCGGTTAGCTCAGTTGGTTAGAGCGTGGTGCTAATAACGCCAAGGTCGCGGGTTCGATCCCCGTACTGGCCA	link

Supplementary Table 4. Multiple t-tests information of Fig. 2g.

Site	Discovery?	P value	Mean of hCtRNA-M	Mean of S	Difference	SE of difference	t ratio	df	q value
1	No	0.006859	36.33	30.67	5.667	1.106	5.126	4	0.017319
2	No	0.053338	60.33	54.33	6	2.211	2.714	4	0.089786
3	No	0.900148	47.33	47.67	-0.3333	2.494	0.1336	4	>0.999999
4	No	0.00559	75.67	69.67	6	1.106	5.427	4	0.017319
5	No	>0.999999	62.33	62.33	0	2.211	0	4	>0.999999

Site	Discovery?	P value	Mean of hCtRNA-M	Mean of P	Difference	SE of difference	t ratio	df	q value
1	No	0.35601	36.33	34.67	1.667	1.599	1.043	4	0.359571
2	No	0.014732	60.33	52	8.333	2.028	4.11	4	0.0186
3	No	0.008335	47.33	40.67	6.667	1.374	4.851	4	0.015618
4	No	0.004813	75.67	70.33	5.333	0.9428	5.657	4	0.015618
5	No	0.009278	62.33	52.67	9.667	2.055	4.704	4	0.015618

Site	Discovery?	P value	Mean of hCtRNA-M	Mean of EF1a-M	Difference	SE of difference	t ratio	df	q value
1	No	0.007467	36.33	28.33	8	1.599	5.004	4	0.015084
2	No	0.025645	60.33	52.67	7.667	2.211	3.467	4	0.025901
3	No	0.019082	47.33	41.67	5.667	1.491	3.801	4	0.025697
4	No	0.121393	75.67	72.33	3.333	1.7	1.961	4	0.098085
5	Yes	0.000583	62.33	36.33	26	2.625	9.906	4	0.002355

Site	Discovery?	P value	Mean of hCtRNA-M	Mean of hU6-M	Difference	SE of difference	t ratio	df	q value
1	No	0.77805	36.33	36.67	-0.3333	1.106	0.3015	4	0.78583
2	No	0.068233	60.33	54.67	5.667	2.285	2.48	4	0.344575
3	No	0.296181	47.33	45	2.333	1.944	1.2	4	0.498571
4	No	0.279083	75.67	73.67	2	1.599	1.251	4	0.498571
5	No	0.462807	62.33	60.67	1.667	2.055	0.8111	4	0.584293

Site	Discovery?	P value	Mean of hCtRNA-M	Mean of w/o 5' leader	Difference	SE of difference	t ratio	df	q value
1	Yes	0.000409	36.33	24.33	12	1.106	10.85	4	0.000298
2	Yes	0.000589	60.33	42	18.33	1.856	9.878	4	0.000298
3	Yes	0.003036	47.33	39.33	8	1.247	6.414	4	0.000767
4	Yes	0.001921	75.67	66	9.667	1.333	7.25	4	0.000647
5	No	0.07712	62.33	57.67	4.667	1.972	2.366	4	0.015578

Site	Discovery?	P value	Mean of hU6-M	Mean of w/o 5' leader	Difference	SE of difference	t ratio	df	q value
1	Yes	0.000587	36.67	24.33	12.33	1.247	9.889	4.000	0.001778
2	Yes	0.001262	54.67	42.00	12.67	1.563	8.102	4.000	0.001912
3	No	0.022270	45.00	39.33	5.667	1.563	3.624	4.000	0.016869
4	No	0.010031	73.67	66.00	7.667	1.667	4.600	4.000	0.010131
5	No	0.053338	60.67	57.67	3.000	1.106	2.714	4.000	0.032323

Site	Discovery?	P value	Mean of EF1a-M	Mean of w/o 5' leader	Difference	SE of difference	t ratio	df	q value
1	No	0.078225	28.33	24.33	4.000	1.700	2.353	4.000	0.047404
2	Yes	0.001833	52.67	42.00	10.67	1.453	7.341	4.000	0.002777
3	No	0.068587	41.67	39.33	2.333	0.9428	2.475	4.000	0.047404
4	No	0.022948	72.33	66.00	6.333	1.764	3.591	4.000	0.023178
5	Yes	0.000414	36.33	57.67	-21.33	1.972	10.82	4.000	0.001255

Site	Discovery?	P value	Mean of EF1a-M	Mean of hU6-M	Difference	SE of difference	t ratio	df	q value

1	No	0.008027	28.33	36.67	-8.333	1.700	4.903	4.000	0.016215
2	No	0.367854	52.67	54.67	-2.000	1.972	1.014	4.000	0.371533
3	No	0.131778	41.67	45.00	-3.333	1.764	1.890	4.000	0.177460
4	No	0.536039	72.33	73.67	-1.333	1.972	0.6761	4.000	0.433119
5	Yes	0.000291	36.33	60.67	-24.33	2.055	11.84	4.000	0.001176

Site	Discovery?	P value	Mean of S	Mean of P	Difference	SE of difference	t ratio	df	q value
1	No	0.078225	30.67	34.67	-4.000	1.700	2.353	4.000	0.131679
2	No	0.234101	54.33	52.00	2.333	1.667	1.400	4.000	0.295552
3	No	0.037542	47.67	40.67	7.000	2.285	3.063	4.000	0.094794
4	No	0.421648	69.67	70.33	-0.6667	0.7454	0.8944	4.000	0.425865
5	No	0.002916	62.33	52.67	9.667	1.491	6.485	4.000	0.014724

Site	Discovery?	P value	Mean of S	Mean of EF1a-M	Difference	SE of difference	t ratio	df	q value
1	No	0.241746	30.67	28.33	2.333	1.700	1.373	4.000	0.244163
2	No	0.426686	54.33	52.67	1.667	1.886	0.8839	4.000	0.344762
3	No	0.063603	47.67	41.67	6.000	2.357	2.546	4.000	0.128477
4	No	0.170618	69.67	72.33	-2.667	1.599	1.668	4.000	0.229766
5	Yes	0.000299	62.33	36.33	26.00	2.211	11.76	4.000	0.001209

Site	Discovery?	P value	Mean of P	Mean of EF1a-M	Difference	SE of difference	t ratio	df	q value
1	No	0.036852	34.67	28.33	6.333	2.055	3.082	4.000	0.074442
2	No	0.709597	52.00	52.67	-0.6667	1.667	0.4000	4.000	0.573355
3	No	0.416866	40.67	41.67	-1.000	1.106	0.9045	4.000	0.421034
4	No	0.250815	70.33	72.33	-2.000	1.491	1.342	4.000	0.337765
5	Yes	0.001357	52.67	36.33	16.33	2.055	7.949	4.000	0.005481

Site	Discovery?	P value	Mean of S	Mean of w/o 5' leader	Difference	SE of difference	t ratio	df	q value
1	No	0.007091	30.67	24.33	6.333	1.247	5.078	4.000	0.014323
2	Yes	0.001056	54.33	42.00	12.33	1.453	8.488	4.000	0.004267
3	No	0.019623	47.67	39.33	8.333	2.211	3.769	4.000	0.026426
4	No	0.037997	69.67	66.00	3.667	1.202	3.051	4.000	0.030701
5	No	0.027391	62.33	57.67	4.667	1.374	3.395	4.000	0.027665

Site	Discovery?	P value	Mean of w/o 5' leader	Mean of P	Difference	SE of difference	t ratio	df	q value
1	Yes	0.003699	24.33	34.67	-10.33	1.700	6.080	4.000	0.005604
2	Yes	0.000978	42.00	52.00	-10.00	1.155	8.660	4.000	0.002964
3	No	0.148148	39.33	40.67	-1.333	0.7454	1.789	4.000	0.089778
4	No	0.014721	66.00	70.33	-4.333	1.054	4.111	4.000	0.011151
5	No	0.010637	57.67	52.67	5.000	1.106	4.523	4.000	0.010743

Site	Discovery?	P value	Mean of hU6-M	Mean of S	Difference	SE of difference	t ratio	df	q value
1	No	0.008581	36.67	30.67	6.000	1.247	4.811	4.000	0.043334
2	No	0.873976	54.67	54.33	0.3333	1.972	0.1690	4.000	0.882716
3	No	0.373901	45.00	47.67	-2.667	2.667	1.000	4.000	0.472050
4	No	0.055041	73.67	69.67	4.000	1.491	2.683	4.000	0.138978
5	No	0.326164	60.67	62.33	-1.667	1.491	1.118	4.000	0.472050

Site	Discovery?	P value	Mean of P	Mean of hU6-M	Difference	SE of difference	t ratio	df	q value
1	No	0.304559	34.67	36.67	-2.000	1.700	1.177	4.000	0.307604
2	No	0.205106	52.00	54.67	-2.667	1.764	1.512	4.000	0.258947
3	No	0.060048	40.67	45.00	-4.333	1.667	2.600	4.000	0.121777
4	No	0.072343	70.33	73.67	-3.333	1.374	2.425	4.000	0.121777

5	No	0.003036	52.67	60.67	-8.000	1.247	6.414	4.000	0.015331

Supplementary Table 5. Protospacers, edited bases (in red) shown in figures, amplicons, and primers. S, supplementary figure.

Figure	Site	Protospacer (5' to 3')	PAM	Benchling
1	CDKN2A	GCCCCAATAATCCCCACATGTCA	TTTA	link
1	DNMT1	TTTCCCTTCAGCTAAAATAAAGG	TTTA	link
1	DYRK1A	GAAGCACATCAAGGACATTCTAA	TTTA	link
1	SITE3	GTTTAAACACACCGGGTTAATAA	TTTG	link
1	VEGFA	CTCTCAAGACCCACAATCCAGGC	TTTG	link
2c,2e,S2,S5,S10	EMX1	GAGTCCGAGCAGAAGAAGAA	GGG	link
2c, 2e, S2, S5	FANCF	GGAATCCCTTCTGCAGCACC	TGG	link
2e, S10	ABE7	GAATACTAAGCATAGACTCC	AGG	link
2e	ABE3	GTCAAGAAAGCAGAGACTGC	CGG	link
2g, S7	1	GTCAAGAAAGCAGAGACTGC	CGG	link
2g, S7	2	GAGCAAAGAGAATAGACTGT	AGG	link
2g, S7	3	GATGAGATAATGATGAGTCA	GGG	link
2g, S7	4	GAATACTAAGCATAGACTCC	AGG	link
2g, S7	5	GAAGATAGAGAATAGACTGC	TGG	link
2h	CDKN2A	CCAATAATCCCCACATGTCA	TGG	link
2h	DNMT1	CCCTTCAGCTAAAATAAAGG	AGG	link
2h	DYRK1A	AGCACATCAAGGACATTCTA	AGG	link
2h	SITE3	TTAAACACACCGGGTTAATA	AGG	link
2h	VEGFA	TCAAGACCCACAATCCAGGC	CGG	link
3a, 3d	1	GAGTCCGAGCAGAAGAAGAA	GGG	link
3a, 3d	2	GGAATCCCTTCTGCAGCACC	TGG	link
3a, 3d	3	GTCATCTTAGTCATTACCTG	AGG	link
3a, 3d	4	GACTCACCCAGGAGTGCGTT	AGG	link
3a, 3d	5	GTATTCACCTGAAAGTGTGC	AGG	link
3a, 3d	6	GAACACAAAGCATAGACTGC	GGG	link
3a, 3d	7	GGCCCAGACTGAGCACGTGA	TGG	link
3a, 3d	8	GGCACTCGGGGGCGAGAGGA	GGG	link
3a, 3d	9	GAGCTCACTGAACGCTGGCA	TGG	link
3a, 3d	10	GCGTGACTTCCACATGAGCG	TGG	link
3b, 3e	1	GTCAAGAAAGCAGAGACTGC	CGG	link
3b, 3e	2	GAGCAAAGAGAATAGACTGT	AGG	link
3b, 3e	3	GATGAGATAATGATGAGTCA	GGG	link
3b, 3e	4	GAATACTAAGCATAGACTCC	AGG	link
3b, 3e	5	GAAGATAGAGAATAGACTGC	TGG	link
3b, 3e	6	GACAAACCAGAAGCCGCTCC	TGG	link
3b, 3e	7	GTTCACACCCATGACGAACA	TGG	link
3b, 3e	8	GAACACAAAGCATAGACTGC	GGG	link
3b, 3e	9	GAAGACCAAGGATAGACTGC	TGG	link
3b, 3e	10	GGTGAGTGAGTGTGTGCGTG	TGG	link
3b, 3e	11	GACAAAGAGGAAGAGAGACG	GGG	link
3b, 3e	12	GAACATAAAGAATAGAATGA	TGG	link

3b, 3e	13	GTAAACAAAGCATAGACTGA	GGG	link
3b, 3e	14	ACACACACACTTAGAATCTG	TGG	link
3b, 3e	15	GAGTATGAGGCATAGACTGC	AGG	link
3b, 3e	16	GGACAGGCAGCATAGACTGT	GGG	link
3b, 3e	17	GTAGAAAAAGTATAGACTGC	AGG	link
3b, 3e	18	GGCTAAAGACCATAGACTGT	GGG	link
3b, 3e	19	GTCTAGAAAGCTTAGACTGC	TGG	link
3b, 3e	20	TCAAGACCCACAATCCAGGC	CGG	link
3c, 3f	1	GACAAAGAGGAAGAGAGACG	GGG	link
3c, 3f	2	GAACATAAAGAATAGAATGA	TGG	link
3c, 3f	3	GTAAACAAAGCATAGACTGA	GGG	link
3c, 3f	4	ACACACACACTTAGAATCTG	TGG	link
3c, 3f	5	GGACAGGCAGCATAGACTGT	GGG	link
3c, 3f	6	GTAGAAAAAGTATAGACTGC	AGG	link
3c, 3f	7	GTCAAGAAAGCAGAGACTGC	CGG	link
3c, 3f	8	GAGCAAAGAGAATAGACTGT	AGG	link
3c, 3f	9	GATGAGATAATGATGAGTCA	GGG	link
3c, 3f	10	GAATACTAAGCATAGACTCC	AGG	link
3c, 3f	11	GAAGATAGAGAATAGACTGC	TGG	link
3c, 3f	12	GACAAACCAGAAGCCGCTCC	TGG	link
3c, 3f	13	GTTCACACCCATGACGAACA	TGG	link
3c, 3f	14	GAACACAAAGCATAGACTGC	GGG	link
3c, 3f	15	GAAGACCAAGGATAGACTGC	TGG	link
3c, 3f	16	GGTGAGTGAGTGTGTGCGTG	TGG	link
3c, 3f	17	CCCTTCAGCTAAAATAAAGG	AGG	link
3c, 3f	18	AGCACATCAAGGACATTCTA	AGG	link
3c, 3f	19	TTAAACACACCGGGTTAATA	AGG	link
3c, 3f	20	GGCTAAAGACCATAGACTGT	GGG	link
3c, 3f	21	GTCTAGAAAGCTTAGACTGC	TGG	link
3c, 3f	22	TCAAGACCCACAATCCAGGC	CGG	link
3c, 3f	23	GAGTCCGAGCAGAAGAAGAA	GGG	link
3c, 3f	24	GGAATCCCTTCTGCAGCACC	TGG	link
3c, 3f	25	GTCATCTTAGTCATTACCTG	AGG	link
3c, 3f	26	GACTCACCCAGGAGTGCGTT	AGG	link
3c, 3f	27	GTATTCACCTGAAAGTGTGC	AGG	link
3c, 3f	28	GAACACAAAGCATAGACTGC	GGG	link
3c, 3f	29	GGCCCAGACTGAGCACGTGA	TGG	link
3c, 3f	30	GGCACTCGGGGGCGAGAGGA	GGG	link
3c, 3f	31	GAGCTCACTGAACGCTGGCA	TGG	link
3c, 3f	32	GCGTGACTTCCACATGAGCG	TGG	link
3j	Sa site1	GTGGTAGACAGCATGTGTCCTA	AAGGGT	link
3j	Sa site2	ATTTACAGCCTGGCCTTTGGGG	TCGGGT	link
3j	Sa site4	GGTGGAGGAGGGTGCATGGGGT	CAGAAT	link
3j	Sa site5	TCTGCTTCTCCAGCCCTGGC	CTGGGT	link
3j	Sa site6	GGATGTTCCAATCAGTACGCA	GAGAGT	link
3k	EMX1 site1	CAGAGTTAGAGCAGAAGAAGAA	AGG	link
3k	EMX1 site2	GAGTCTAAGCAGAAGAAGAA	GAG	link

3k	EMX1 site3	GAGGCCGAGCAGAAGAAAGA	CGG	link
3k	EMX1 site4	GAGTCCTAGCAGGAGAAGAA	GAG	link
3k	EMX1 site5	AAGTCTGAGCACAAGAAGAA	TGG	link
3k	EMX1 site6	GAGTCCGGGAAGGAGAAGAA	AGG	link
3k	EMX1 site10	ACGTCTGAGCAGAAGAAGAA	TGG	link
S14	1	GTCAAGAAAGCAGAGACTGC	CGG	link
S14	2	GAGCAAAGAGAATAGACTGT	AGG	link
S14	3	GATGAGATAATGATGAGTCA	GGG	link
S14	4	GAATACTAAGCATAGACTCC	AGG	link
S14	5	GAAGATAGAGAATAGACTGC	TGG	link
S14	6	GACAAACCAGAAGCCGCTCC	TGG	link
S14	7	GTTCACACCCATGACGAACA	TGG	link
S14	8	GAACACAAAGCATAGACTGC	GGG	link
S14	9	GAAGACCAAGGATAGACTGC	TGG	link
S14	10	GGTGAGTGAGTGTGTGCGTG	TGG	link
4a, 4b	HEK3 (P)	GGCCCAGACTGAGCACGTGA	TGG	link
4a, 4b	HEK3 (N)	GTCAACCAGTATCCCGGTGC	AGG	link
4a, 4b	RNF2 (P)	GTCATCTTAGTCATTACCTG	AGG	link
4a, 4b	RNF2 (N)	TCAACCATTAAGCAAAACAT	GGG	link
4a, 4b	FANCF (P)	GGAATCCCTTCTGCAGCACC	TGG	link
4a, 4b	FANCF (N)	GGGGTCCCAGGTGCTGACGT	AGG	link
5a, S21	APOC3 D65N	GCCATCGGTCACCCAGCCCC	TAA	link
5a, S21	APOC3 IVS+1	GCTTACGGGCAGAGGCCAGG	AGC	link
5a, S21	MSTN	GACTACTTACACTCTGTAGG	CAT	link
5a, S21	SLC30A8	GGTACATGCCTCCTGACTCC	AGC	link
5c	BCL11A (target 2)	TTTATCACAGGCTCCAGGAA	GGG	link
5c	HBG1/2	CTTGACCAATAGCCTTGACA	AGG	link
5j, 5k, S28	BCL11A	TTTATCACAGGCTCCAGGAA	GGG	link
5e-h	BCL11A	CTAACAGTTGCTTTTATCAC	AGG	link
5e-h	HBG1	CTTGACCAATAGCCTTGACA	AGG	link
5e-h	HBG2	CTTGACCAATAGCCTTGACA	AGG	link
S22	BCL11A (target 1)	CTAACAGTTGCTTTTATCAC	AGG	link
S22	BCL11A (target 2)	TTTATCACAGGCTCCAGGAA	GGG	link
S22	HBG1	CTTGACCAATAGCCTTGACA	AGG	link
S22	HBG2	CTTGACCAATAGCCTTGACA	AGG	link
S5	RNF2	GTCATCTTAGTCATTACCTG	AGG	link
S5	PPP1R12C	GACTCACCCAGGAGTGCGTT	AGG	link
S12	1	GAGTCCGAGCAGAAGAAGAA	GGG	link
S12	2	GGAATCCCTTCTGCAGCACC	TGG	link
S12	3	GTCATCTTAGTCATTACCTG	AGG	link
S12	4	GACTCACCCAGGAGTGCGTT	AGG	link
S12	5	GTATTCACCTGAAAGTGTGC	AGG	link
S12	6	GAACACAAAGCATAGACTGC	GGG	link
S12	7	GGCCCAGACTGAGCACGTGA	TGG	link
S12	8	GGCACTCGGGGGCGAGAGGA	GGG	link
S12	9	GAGCTCACTGAACGCTGGCA	TGG	link
S12	10	GCGTGACTTCCACATGAGCG	TGG	link

S13b	1	GTCAAGAAAGCAGAGACTGC	CGG	link
S13b	2	GAGCAAAGAGAATAGACTGT	AGG	link
S13b	3	GATGAGATAATGATGAGTCA	GGG	link
S13b	4	GAATACTAAGCATAGACTCC	AGG	link
S13b	5	GAAGATAGAGAATAGACTGC	TGG	link
S13b	6	GACAAACCAGAAGCCGCTCC	TGG	link
S13b	7	GTTCACACCCATGACGAACA	TGG	link
S13b	8	GAACACAAAGCATAGACTGC	GGG	link
S13b	9	GAAGACCAAGGATAGACTGC	TGG	link
S13b	10	GGTGAGTGAGTGTGTGCGTG	TGG	link
S13c, S15, S17	1	GAGTCCGAGCAGAAGAAGAA	GGG	link
S13c, S15, S17	2	GGAATCCCTTCTGCAGCACC	TGG	link
S13c, S15, S17	3	GTCATCTTAGTCATTACCTG	AGG	link
S13c, S15, S17	4	GACTCACCCAGGAGTGCGTT	AGG	link
S13c, S15, S17	5	GTATTCACCTGAAAGTGTGC	AGG	link
S13c, S15, S17	6	GAACACAAAGCATAGACTGC	GGG	link
S13c, S15, S17	7	GGCCCAGACTGAGCACGTGA	TGG	link
S13c, S15, S17	8	GGCACTCGGGGGCGAGAGGA	GGG	link
S13c, S15, S17	9	GAGCTCACTGAACGCTGGCA	TGG	link
S13c, S15, S17	10	GCGTGACTTCCACATGAGCG	TGG	link
S18	1	GTCAAGAAAGCAGAGACTGC	CGG	link
S18	2	GAGCAAAGAGAATAGACTGT	AGG	link
S18	3	GATGAGATAATGATGAGTCA	GGG	link
S18	4	GAATACTAAGCATAGACTCC	AGG	link
S18	5	GAAGATAGAGAATAGACTGC	TGG	link
S18	6	GACAAACCAGAAGCCGCTCC	TGG	link
S18	7	GTTCACACCCATGACGAACA	TGG	link
S18	8	GAACACAAAGCATAGACTGC	GGG	link
S18	9	GAAGACCAAGGATAGACTGC	TGG	link
S18	10	GGTGAGTGAGTGTGTGCGTG	TGG	link
S18	11	GACAAAGAGGAAGAGAGACG	GGG	link
S18	12	GAACATAAAGAATAGAATGA	TGG	link
S18	13	GTAAACAAAGCATAGACTGA	GGG	link
S18	14	ACACACACACTTAGAATCTG	TGG	link
S18	15	GAGTATGAGGCATAGACTGC	AGG	link
S18	16	GGACAGGCAGCATAGACTGT	GGG	link
S18	17	GTAGAAAAAGTATAGACTGC	AGG	link
S18	18	GGCTAAAGACCATAGACTGT	GGG	link
S18	19	GTCTAGAAAGCTTAGACTGC	TGG	link
S18	20	TCAAGACCCACAATCCAGGC	CGG	link
S19a	BCL11A	TTTATCACAGGCTCCAGGAA	GGG	link
S19a	HBG1	CTTGACCAATAGCCTTGACA	AGG	link
S19a	HBG2	CTTGACCAATAGCCTTGACA	AGG	link
S19c	1	GACAAAGAGGAAGAGAGACG	GGG	link
S19c	2	GAACATAAAGAATAGAATGA	TGG	link
S19c	3	GTAAACAAAGCATAGACTGA	GGG	link
S19c	4	ACACACACACTTAGAATCTG	TGG	link

S19c	5	GGACAGGCAGCATAGACTGT	GGG	link
S19c	6	GTAGAAAAAGTATAGACTGC	AGG	link
S19c	7	GTCAAGAAAGCAGAGACTGC	CGG	link
S19c	8	GAGCAAAGAGAATAGACTGT	AGG	link
S19c	9	GATGAGATAATGATGAGTCA	GGG	link
S19c	10	GAATACTAAGCATAGACTCC	AGG	link
S19c	11	GAAGATAGAGAATAGACTGC	TGG	link
S19c	12	GACAAACCAGAAGCCGCTCC	TGG	link
S19c	13	GTTCACACCCATGACGAACA	TGG	link
S19c	14	GAACACAAAGCATAGACTGC	GGG	link
S19c	15	GAAGACCAAGGATAGACTGC	TGG	link
S19c	16	GGTGAGTGAGTGTGTGCGTG	TGG	link
S19c	17	CCCTTCAGCTAAAATAAAGG	AGG	link
S19c	18	AGCACATCAAGGACATTCTA	AGG	link
S19c	19	TTAAACACACCGGGTTAATA	AGG	link
S19c	20	GGCTAAAGACCATAGACTGT	GGG	link
S19c	21	GTCTAGAAAGCTTAGACTGC	TGG	link
S19c	22	TCAAGACCCACAATCCAGGC	CGG	link
S19c	23	GAGTCCGAGCAGAAGAAGAA	GGG	link
S19c	24	GGAATCCCTTCTGCAGCACC	TGG	link
S19c	25	GTCATCTTAGTCATTACCTG	AGG	link
S19c	26	GACTCACCCAGGAGTGCGTT	AGG	link
S19c	27	GTATTCACCTGAAAGTGTGC	AGG	link
S19c	28	GAACACAAAGCATAGACTGC	GGG	link
S19c	29	GGCCCAGACTGAGCACGTGA	TGG	link
S19c	30	GGCACTCGGGGGCGAGAGGA	GGG	link
S19c	31	GAGCTCACTGAACGCTGGCA	TGG	link
S19c	32	GCGTGACTTCCACATGAGCG	TGG	link
S23a, S24	BCL11A	TTTATCACAGGCTCCAGGAA	GGG	link
S23a, S24	HBG1	CTTGACCAATAGCCTTGACA	AGG	link
S23c	BCL11A	CTAACAGTTGCTTTTATCAC	AGG	link
S23c	HBG1	CTTGACCAATAGCCTTGACA	AGG	link
S23c	HBG2	CTTGACCAATAGCCTTGACA	AGG	link
S23e	EMX1	GAGTCCGAGCAGAAGAAGAA	GGG	link
S23e	FANCF	GGAATCCCTTCTGCAGCACC	TGG	link
S23e	PPP1R12C	GACTCACCCAGGAGTGCGTT	AGG	link
S23g, S23i	BCL11A	TTTATCACAGGCTCCAGGAA	GGG	link
S23g, S23i	HBG1	CTTGACCAATAGCCTTGACA	AGG	link
S23g, S23i	HBG2	CTTGACCAATAGCCTTGACA	AGG	link
S3	EMX1	GAGTCCGAGCAGAAGAAGAA	GGG	link
S3	RNF2	GTCATCTTAGTCATTACCTG	AGG	link
S3	HEK3	GGCCCAGACTGAGCACGTGA	TGG	link
S3	FANCF	GGAATCCCTTCTGCAGCACC	TGG	link
S3	PPP1R12C	GACTCACCCAGGAGTGCGTT	AGG	link
S4	FANCF	GGAATCCCTTCTGCAGCACC	TGG	link
S4	EMX1	GAGTCCGAGCAGAAGAAGAA	GGG	link
S4	RNF2	GTCATCTTAGTCATTACCTG	AGG	link

S6	ABE site3	GTCAAGAAAGCAGAGACTGC	CGG	link
S6	ABE site4	GAGCAAAGAGAATAGACTGT	AGG	link
S6	ABE site5	GATGAGATAATGATGAGTCA	GGG	link
S6	ABE site7	GAATACTAAGCATAGACTCC	AGG	link
S6	ABE site13	GAAGATAGAGAATAGACTGC	TGG	link
S9	ABE site3	GTCAAGAAAGCAGAGACTGC	CGG	link
S9	ABE site4	GAGCAAAGAGAATAGACTGT	AGG	link
S9	ABE site5	GATGAGATAATGATGAGTCA	GGG	link
S9	ABE site7	GAATACTAAGCATAGACTCC	AGG	link
S9	ABE site13	GAAGATAGAGAATAGACTGC	TGG	link
S16a	1	GTCAAGAAAGCAGAGACTGC	CGG	link
S16a	2	GAGCAAAGAGAATAGACTGT	AGG	link
S16a	3	GATGAGATAATGATGAGTCA	GGG	link
S16a	4	GAATACTAAGCATAGACTCC	AGG	link
S16a	5	GAAGATAGAGAATAGACTGC	TGG	link
S16a	6	GACAAACCAGAAGCCGCTCC	TGG	link
S16a	7	GTTCACACCCATGACGAACA	TGG	link
S16a	8	GAACACAAAGCATAGACTGC	GGG	link
S16a	9	GAAGACCAAGGATAGACTGC	TGG	link
S16a	10	GGTGAGTGAGTGTGTGCGTG	TGG	link
S16b	1	GAGTCCGAGCAGAAGAAGAA	GGG	link
S16b	2	GGAATCCCTTCTGCAGCACC	TGG	link
S16b	3	GTCATCTTAGTCATTACCTG	AGG	link
S16b	4	GACTCACCCAGGAGTGCGTT	AGG	link
S16b	5	GTATTCACCTGAAAGTGTGC	AGG	link
S16b	6	GAACACAAAGCATAGACTGC	GGG	link
S16b	7	GGCCCAGACTGAGCACGTGA	TGG	link
S16b	8	GGCACTCGGGGGCGAGAGGA	GGG	link
S16b	9	GAGCTCACTGAACGCTGGCA	TGG	link
S16b	10	GCGTGACTTCCACATGAGCG	TGG	link
S25	FANCF	GGAATCCCTTCTGCAGCACC	TGG	link
S25	PPP1R12C	GACTCACCCAGGAGTGCGTT	AGG	link

References

1. Lee S*, et al.* Single C-to-T substitution using engineered APOBEC3G-nCas9 base editors with minimum genome-and transcriptome-wide off-target effects. **6**, eaba1773 (2020).

2. Conant D*, et al.* Inference of CRISPR Edits from Sanger Trace Data. *The CRISPR Journal* **5**, 123-130 (2022).

3. DeWeirdt PC*, et al.* Optimization of AsCas12a for combinatorial genetic screens in human cells. *Nature Biotechnology* **39**, 94-104 (2021).

4. Nuñez JK*, et al.* Genome-wide programmable transcriptional memory by CRISPR-based epigenome editing. *Cell* **184**, 2503-2519 (2021).
